# The Biological Consequences of the Knockout of Genes Involved in the Synthesis and Metabolism of H_2_S in *Drosophila melanogaster*

**DOI:** 10.3390/antiox14060693

**Published:** 2025-06-06

**Authors:** Victoria Y. Shilova, David G. Garbuz, Lyubov N. Chuvakova, Alexander P. Rezvykh, Sergei Y. Funikov, Artem I. Davletshin, Svetlana Y. Sorokina, Ekaterina A. Nikitina, Olga Gorenskaya, Michael B. Evgen’ev, Olga G. Zatsepina

**Affiliations:** 1 Engelhardt Institute of Molecular Biology, Russian Academy of Sciences, 119991 Moscow, Russia; vika-shilova@yandex.ru (V.Y.S.); dgarbuz@yandex.ru (D.G.G.); lyubov.chuvakova@gmail.com (L.N.C.); aprezvykh@yandex.ru (A.P.R.); sergeifunikov@mail.ru (S.Y.F.); artem.dav7@yandex.ru (A.I.D.); olgavg2014@gmail.com (O.G.); misha672011@yahoo.com (M.B.E.); 2 Koltzov Institute of Developmental Biology, Russian Academy of Sciences, 119334 Moscow, Russia; svetlana_ibr@mail.ru; 3 Pavlov Institute of Physiology, Russian Academy of Sciences, 199034 Saint-Petersburg, Russia; 21074@mail.ru; 4 Herzen State Pedagogical University of Russia, Biology Department, 191186 Saint-Petersburg, Russia; 5 Federal Scientific Center of Hygiene Named After F.F. Erisman of the Federal Service for Surveillance on Consumer Rights Protection and Human Wellbeing, 141014 Mytishchi, Russia; 6 Institute of Evolution, University of Haifa, Haifa 3498838, Israel

**Keywords:** *Drosophila melanogaster*, knockout (KO) strains, cystathionine-β-synthase, cystathionine γ-lyase, sulfurtransferase, life-history traits

## Abstract

Here, we describe the effects of double knockout (KO) of the *cbs* and *cse* genes, which are responsible for H_2_S synthesis through the transsulfuration pathway, and KO of the sulfurtransferase gene (*dtst1*) in *Drosophila melanogaster* females. The analysis of H_2_S production in flies showed minimal levels in the double- and triple-knockout strains. The double- (*cbs-/-*; *cse-/-*) and triple- (*cbs-/-*; *cse-/-*; *dtst-/-*) KO flies exhibited a shortened lifespan and reduced fecundity, and showed dramatic changes in Malpighian tubule morphology. The transcriptomic analysis revealed a profound increase in the expression levels of several genes involved in excretory system function in the double-KO and especially the triple-KO flies. Importantly, major groups of differentially expressed genes (DEGs) in the whole bodies of females and ovaries of KO strains included genes responsible for detoxification, reproduction, mitochondrial activity, excretion, cell migration, and muscle system function. The reduced fecundity observed in the double- and triple-KO flies correlated with pronounced changes in the ovarian transcriptome. At the same time, the single knockout of *dtst1* increased the flies’ fecundity and lifespan. Our experiments exploring unique *Drosophila* strains with KO of major H_2_S-related genes revealed several new pathways controlled by this ancient adaptogenic system that is involved in various human diseases and aging.

## 1. Introduction

Two decades ago, hydrogen sulfide (H_2_S), the simplest of the thiols found in the cells of different organisms from bacteria to mammals, joined the other two well-known gases, carbon monoxide (CO) and nitric oxide (NO), to become the third essential gasotransmitter [1,2,3]. Three enzymes, cystathionine-β-synthase, cystathionine γ-lyase, and 3-mercaptopyruvate sulfurtransferase (CBS, CSE, and 3-MST), are involved in the endogenous production of H_2_S in various cells and organs [4,5,6,7,8,9] through direct or indirect use of sulfur-containing amino acids, although a certain proportion of H_2_S is produced via non-enzymatic reactions [10,11,12].

It was demonstrated in numerous studies that H_2_S and other reactive sulfur species play a key role in different cellular regulatory processes, including inflammation, energy metabolism, neurotransmission, redox signaling, etc. [13,14]. At the same time, using *cbs/cse/3-mst* triple-KO mice, it was shown that these genes are not significant sources of endogenous reactive persulfide production in mammals [15].

Notably, CBS and CSE have crucial functions in the metabolism of sulfur-containing amino acids in the transsulfuration pathway (TSP). The KO of these genes is detrimental for an organism because it leads to the accumulation of toxic TSP intermediates, i.e., homocysteine and cystathionine, and affects methionine levels as well as the products of the methyltransferase pathway [16].

The significance of TSP genes in the pathophysiology of various diseases, including neurodegenerative disorders, and recent advances in developing efficient therapies targeting this pathway have been summarized in several reviews [17,18,19,20]. Importantly, H_2_S also plays a crucial role in various physiological processes related to the functioning of the female reproductive system in mammals, including humans [21,22].

The universality and high conservation of the ancient adaptogenic system controlling H_2_S synthesis and metabolism in all organisms from bacteria to humans make *Drosophila* flies an ideal model for studying the role of both the whole H_2_S system and its components in various biological functions, from reproduction and longevity to resistance to bacterial infection. One convenience of using *Drosophila* is the possibility of obtaining deletion transformants with either a single gene deletion or combined (double and triple) knockouts. This enables the identification of the contribution of each of these genes to physiological processes.

Previously, using CRISPR-Cas9 technology, we obtained deletions of the *cbs* (*CG1753*) and *cse* (*CG5345*) genes and studied the effects of these deletions on several vital fitness characteristics of *D. melanogaster* flies [23,24,25]. Moreover, we described the impact of single and double KOs of *cbs* and *cse* genes on the transcriptome of males and females [26]. 

The third important member of the TSP is 3-mercaptopyruvate sulfurtransferase (3-MST). 3-MST, a member of the sulfurtransferase superfamily, participates in the production of H_2_S as well as in detoxification reactions in the cytoplasm and mitochondria in most eukaryotic organisms, including mammals [27,28,29]. Another gene belonging to the same family encodes a thiosulfate sulfurtransferase (TST), known as “rhodanese”. It detoxifies cyanide (CN−) by converting it to thiocyanate in mitochondria. It catalyzes the transfer of sulfur from GSSH to sulfite-forming GSH and thiosulfate and, hence, represents a component of the mitochondrial pathway of sulfide oxidation [30]. Importantly, 3-MST and rhodanese (TST) enzymes can both detoxify cyanide (CN^−^) and produce H_2_S [31].

While a plethora of studies described the vital role of 3-MST and TST in mammals and other organisms [32], neither enzyme has been detected in *Drosophila* [29]. However, in the genomes of eukaryotes, there is another family that includes single-domain sulfurtransferases that can perform similar functions in the cell [32]. To date, the most studied single-domain sulfurtransferase in humans is TSTD1, which, like the TST and MST enzymes, can catalyze the transfer of sulfur to cyanide and thioredoxin [33]. It was hypothesized that TSTD1 functions as a sulfur shuttle between donor and acceptor proteins and may play a role in sulfide-based signaling and cyanide detoxification [33]. 

It was also proposed that TSTD1 in humans is involved in the sulfide oxidative pathway and H_2_S catabolism [34].

Previously, based on the available sequencing data, we inactivated the *CG12279* gene, which encodes a protein with a single rhodanese domain, in *D. melanogaster* using CRISPR/Cas9 technology [26]. The nucleotide sequence analysis demonstrated that this gene (*CG12279*) is orthologous to the human *tstd1* gene. Based on these data, we designated the *CG12279* gene as *Drosophila tst1* or “*dtst1*” in this paper.

Since the specific physiological roles for most of the sulfurtransferases are currently unknown [33,35,36], it was interesting to study the effect of *dtst1* KO on different physiological aspects of *Drosophila* biology and to study the interactions between *dtst1* and the major TSP genes, i.e., *cbs* and *cse,* that are responsible for H_2_S synthesis in flies.

The available data allowed us to define two alternative hypotheses regarding the interaction between *dtst1* and the genes involved in the TSP. If the main function of dTST1 is H_2_S utilization in mitochondria, its knockout should partially attenuate the effect of the double deletion of the *cbs* and *cse* genes, restoring the level of this gas in the organism by slowing down the rate of its degradation. In this case, the strain with a single deletion of *dtst1* should also demonstrate an increase in H_2_S production. However, if the main function of *dtst1* is to transfer sulfane sulfur from protein to protein and, consequently, to participate in sulfide-based signaling, then its deletion may exacerbate the effect of deletions of transsulfuration genes at both the physiological and transcriptomic levels. Notably, if *dtst1* is involved in cyanide detoxification, its deletion may cause an increase in cyanide levels in the body. The increase in cyanide level on the background of deletions of the *cbs* and *cse* genes may increase intoxication of the organism, which will lead to overload of the excretory system in the *Drosophila*.

In the present study, we measured H_2_S levels in the generated KO strains and demonstrated that KO of the *cbs* and *cse* genes can reduce the level of this gas in the bodies of adult flies, especially in the double- and triple-KO strains. The reduced level of H_2_S in these strains resulted in significant effects on several life-history traits, including lifespan and certain reproduction parameters. Importantly, the KOs drastically influenced the transcription of numerous loci in the genome, including genes responsible for detoxification, reproduction, excretion, and mitochondrial function. At the same time, single deletion of the *dtst1* gene improved physiological parameters such as reproductive capacity and lifespan. All the results were obtained from female *Drosophila* since the main effect of deletion of the *cbs* and *cse* genes is reduced fecundity and structural abnormalities in the female reproductive system. Therefore, we took full advantage of the *Drosophila* model to elucidate the biological functions of H_2_S.

## 2. Materials and Methods

### 2.1. Drosophila melanogaster Strains

The development of transgenic knockout (KO) strains bearing CRISPR/Cas9-mediated deletions of cystathionine ß-synthase (*cbs*), cystathionine γ-lyase (*cse*), and *CG12279* genes has been described previously. Only knockout strains without off-targets, which was confirmed by Southern hybridization, were selected for the experiments [26]. Strain 58492 (BDSC_58492, Bloomington, IA, USA), which was previously used to generate the *cbs* and *cse* deletions, was used as the control strain [26]. All the strains were maintained, and the experiments were conducted under constant environmental conditions (25 °C, 60% relative humidity, and 12 h light/dark cycle), which were provided by a Binder KBF 720 climate chamber (Binder, Tuttlingen, Germany).

### 2.2. Detection of H_2_S Production Level in Fly Samples

Twenty-five seven-day-old females from each strain were starved for six hours; approximately 30 mg of tissue from these flies was homogenized in 150 μL of PBS containing 10 mM L-cysteine, 1 mM pyridoxal 5′-phosphate hydrate (PLP), a protease inhibitor cocktail (cOmplete^TM^, EDTA-free Protease Inhibitor Cocktail; Roche), and 50 µg/mL ampicillin [37]. The homogenates were transferred to a 96-well plate. Paper with 5% lead acetate was placed on top of the plate. The plate was incubated at 30 °C for 20 h, followed by imaging and analysis of the dark circles that appeared on the filter paper due to the formation of lead sulfide. The optical density of the circles was measured using the ImageJ software, version 1.53e. The results of three experiments were taken into account, and the errors of the experiment were calculated using Student’s *t* test.

### 2.3. Lifespan Analysis

Flies from the control and experimental strains were collected in vials on the day of eclosion. Using a carbon dioxide anesthetizing apparatus (Genesee Scientific, El Cajon, CA, USA), the flies were sorted by sex and placed in vials containing food medium at a density of 25 flies per vial. Twelve vials per group were analyzed. The food medium on which the control and experimental flies were maintained during all the experiments was prepared according to the formulation described in [38]. The number of dead flies was counted daily starting from the first day of the imago stage. The food vials were replaced with fresh ones two times per week. All the experiments were performed at least three times. The median (50%) and maximum lifespans (90% death) were estimated for all the strains.

Statistical analysis was performed using an online application for survival analysis, OASIS 2 [39]. Kaplan–Meier curves were used to graphically represent the survival. The log-rank test was used to compare the survival curves. The statistical significance of the differences at the 50th (median lifespan) and 90th percentiles of lifespan was assessed using the log-rank test with Bonferroni correction and the Wang–Allison (Boschloo’s) test [40], respectively.

### 2.4. Analysis of the Reproductive Parameters

For the analysis of fertility dynamics, newly eclosed virgin females were individually crossed with two males. All flies of each experimental group, consisting of ten females, were transferred to vials with fresh medium every 24 h. The number of laid eggs was counted under an SZX10 stereomicroscope (Olympus, Hachioji, Japan). The kinetics of oviposition was estimated as the mean number of embryos produced by a single female per day for the first 8 days. After egg counting, the vials were kept to determine the proportion of pupated larvae and eclosed flies.

For the analysis of the reproductive period, 12 newly eclosed virgin females were individually crossed with two males. Every three days, the flies were transferred to a new vial. The number of eclosed flies was counted in each vial during the whole reproductive period. The results of three experiments were taken into account. Significance (* *p* < 0.05) was determined by two-way ANOVA followed by post hoc Tukey’s HSD tests. The error of the experiment was calculated using Student’s *t* test.

### 2.5. Fluorescent Staining of the Ovaries

Ovaries were dissected from 4-day-old flies, washed in a PBS solution, and fixed in 4% paraformaldehyde (PFA) diluted in 1× PBS for 20 min; then, they were washed 4 times in PBS and 20% Triton. F-actin staining was performed by incubating the ovaries in 1 unit of Phalloidin–Atto Rho6G (Cat. No. R415; Thermo Fisher Scientific, Waltham, MA, USA) for 1 h at room temperature. The ovaries were washed in PBS and treated with RNAse (final concentration: 250 μg/mL) at +37 °C for 20 min, followed by washes in PBS. Nuclei staining was performed using Sytox Green nucleic acid stain (Thermo Fisher Scientific Cat. No S7020, Waltham, MA, USA) at a 1:300 dilution for 10 min at room temperature. After washes in PBS, the ovaries were mounted in Fluoroshield solution. Fluorescent images were obtained using a confocal microscope.

### 2.6. The Generation of Triple-Knockout Flies (cbs, cse, and dtst1)

To obtain triple-KO flies, we used an available double-KO strain for the *cbs* and *cse* genes and flies from a strain with a knockout of the *CG12279* gene, which was designated as “*dtst1*” in this paper [26]. At the first stage, we used the balancer strain w, CyO/L; Tb/Sb to cross with *cbs-/-*; *cse-/-* to obtain a *cbs-/-*; *cse-/-*; HT/Sb strain. In the case of the *dtst1-/-* strain, we used the balancer strain w; CyO/L; D/Sb and obtained a *CG12279-/-*: w; CyO/L strain. At the next stage, we combined these strains to obtain triple-KO flies with inactivated *cbs*, *cse*, and *dtst1* genes. To confirm the inactivation of these genes (*cbs*, *cse*, and *dtst1*), we performed real-time PCR (Figure S1).

### 2.7. RNA Extraction and Quantitative Real-Time PCR

The procedures used were identical to those described in [24]. Briefly, total RNA was extracted from adult five-day-old flies or ovaries from control 58492 and knockout strains using guanidine isothiocyanate RNAzol RT (Molecular Research Center, Cincinnati, OH, USA) following the manufacturer’s protocol. All experiments were performed with three to five biological replicates and three experimental replicates. The primers used in the qRT-PCR experiments are given in Table S1.

### 2.8. RNA-Seq Library Preparation and Transcriptomic Analysis

Total RNA extraction from whole adult female flies and ovaries was performed as described in the previous section. The RNA-seq bioinformatic analysis was performed using the standard steps of read trimming using Trimmomatic [41], read alignment to reference genome (BDGP 6.54, Ensembl) using STAR aligner [42], and counting reads spanning into exons of protein-coding genes using featureCounts [43]. Differential expression analysis was implemented in the PPLine pipeline [44] and performed with the edgeR package [45], with an FDR < 0.05 set as the significance cutoff for differentially expressed genes. GSEAs were conducted using the ShinyGO v.77 and clusterProfiler packages [46,47]. Visualization of the transcriptomic data was performed using the ggplot2, gplots, and pheatmap R packages. RNA sequencing was performed using the equipment of the Engelhardt Institute of Molecular Biology RAS ‘Genome’ Centre (http://www.eimb.ru/rus/ckp/ccu_genome_c.php (accessed on 15 February 2025)) [26]. The sequence data were deposited in the NCBI GEO database under accession numbers GSE279189 and GSE148109.

### 2.9. Malpighian Tubule Dissection and Staining

MTs from five-day-old females from all the investigated strains were dissected in PBS and labeled with 2.5 μM SF7-AM in PBS for 30 min, washed 3 times with PBS, and mounted in PBS. The live-cell imaging and analysis were performed using an Evos FL microscope at a magnification of 4× or 10×. MT measurements were performed using the ImageJ software, version 1.53e. At least three MTs were analyzed for each strain. The average size was calculated.

## 3. Results

### 3.1. H_2_S Production Is Drastically Reduced in Double- and Triple-Knockout Strains

**Figure 1:**
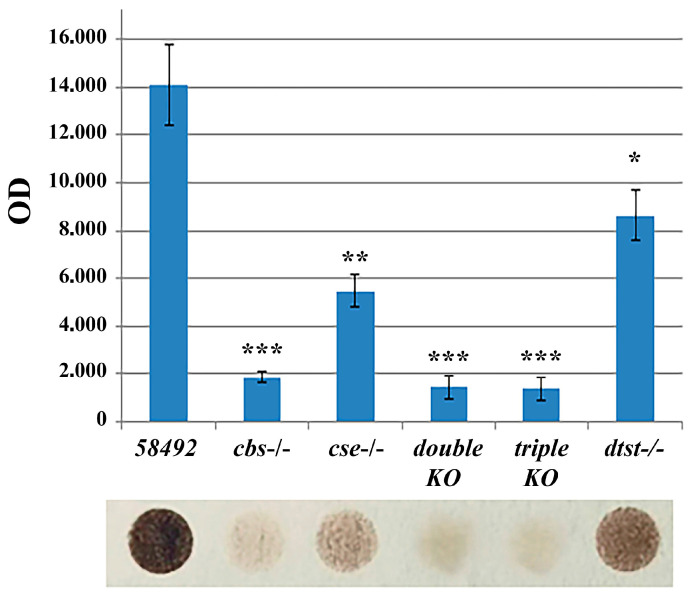
Detection of H_2_S production in extracts from different *Drosophila* strains. H_2_S production in the extracts from the bodies of seven-day-old flies in the presence of L-cysteine and PLP. H_2_S production was measured using the acetate/lead sulfide method (M@M). The results of the five experiments were taken into account. * *p* < 0.05, ** *p* < 0.01, *** *p* < 0.001 (vs. control 58492 flies). Statistical significance was calculated using Student’s *t* test.

It is currently unclear in which physiological processes in *Drosophila* the sulfurtransferase (*tst*) genes are involved in and how they interact with key genes of the transsulfuration pathway, i.e., *cbs* and *cse*. In the present study, we combined a strain with an inactivated *dtst1* gene with a strain containing a double deletion of the *cbs* and *cse* genes through genetic methods and obtained triple-KO flies (*cbs-/-*; *cse-/-*; *dtst1-/-*). Since there are non-enzymatic pathways for H_2_S metabolism and synthesis [10,48], the first step in our work was to investigate whether the synthesis of this transmitter is altered in flies with knockouts of the genes involved in H_2_S synthesis and/or metabolism. The efficiency of the generated knockouts was determined using the RT PCR technique (Figure S1). Since the microbiota can contribute markedly to H_2_S production [49], to reduce its influence, we subjected the flies to a six-hour fasting period before preparing the extracts (Figure 1). The H_2_S production in body extracts from seven-day-old flies of all the strains was measured by the lead acetate/sulfide method (Section 2). The results of three experiments were taken into account. Calibration was performed using the NaHS dilutions shown in Figure S2.

Figure 1 shows that all the strains with deletions had lower levels of H_2_S synthesis in the whole body compared to control flies. The lowest level of synthesis was observed in the *cbs-/-,* double-, and triple-KO strains. The level of hydrogen sulfide production in the double- and triple-knockout strains was so low that it could barely be detected. In the strains with KO of the *dtst1* gene, H_2_S production was also significantly reduced but remained at a fairly high level. This suggests that dTST1 is most likely not engaged in the degradation of hydrogen sulfide in mitochondria [34] but is probably involved in sulfur transfer processes [33].

### 3.2. Genes Involved in H_2_S Synthesis Affect the Lifespan of Drosophila Females

**Figure 2:**
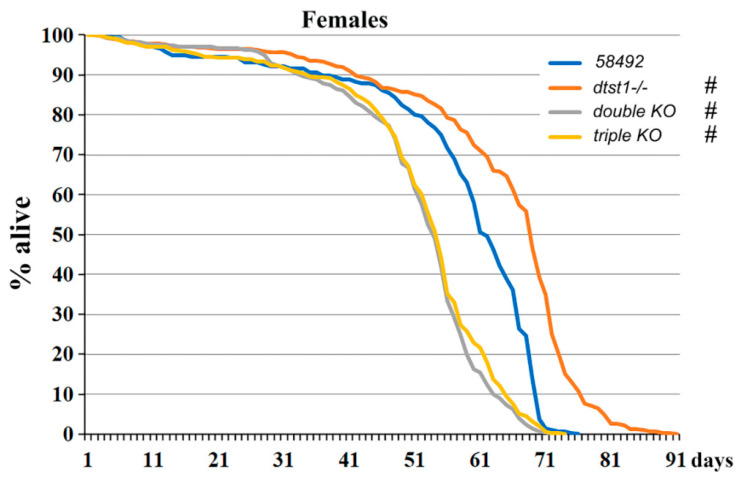
Lifespan of KO strains. Effects of *dtst1* KO, and double- and triple-KOs on lifespan of *Drosophila* females # *p* < 0.001 (vs. control 58492 flies). Significance was determined using the log-rank test with Bonferroni corrections. Lifespan data were pooled from three independent experiments.

Since genes involved in the TSP are known to affect multiple major biological systems in animals, it was of interest to investigate how inactivation of individual components of this system, as well as double- and triple-KO of these genes, affected the lifespan of females. It was previously shown [25] that deletion of the *cbs* gene decreases the lifespan of flies while deletion of the *cse* gene increases it. In the present work, we extended these studies and examined the effects of *dtst1* gene inactivation, as well as the effects of double and triple KOs on the lifespan of *Drosophila*. The original strain *58492*, from which all knockout strains were obtained, was used as the control (Figure 2). The results suggest that KO of *dtst1* significantly increases the lifespan of females compared to the control flies. The median and maximum lifespan of *dtst1-/-* females increased by 11% (Table S2). Interestingly, the lifespan of the double- and triple-KO flies, which had the lowest H_2_S levels (Figure 1), were similar and significantly shorter than that of the control flies. The median lifespan of the double-KO females was 9% shorter than that of the control flies, and the maximum lifespan was 10% shorter. The median lifespan in the triple-KO females was 11.5% shorter than that of the control flies, and the maximum lifespan was 7% shorter.

To understand the processes involved in the differences in lifespan in the flies with deletions, we performed transcriptome analysis of whole flies to identify differentially expressed genes between the strains.

**Figure 3:**
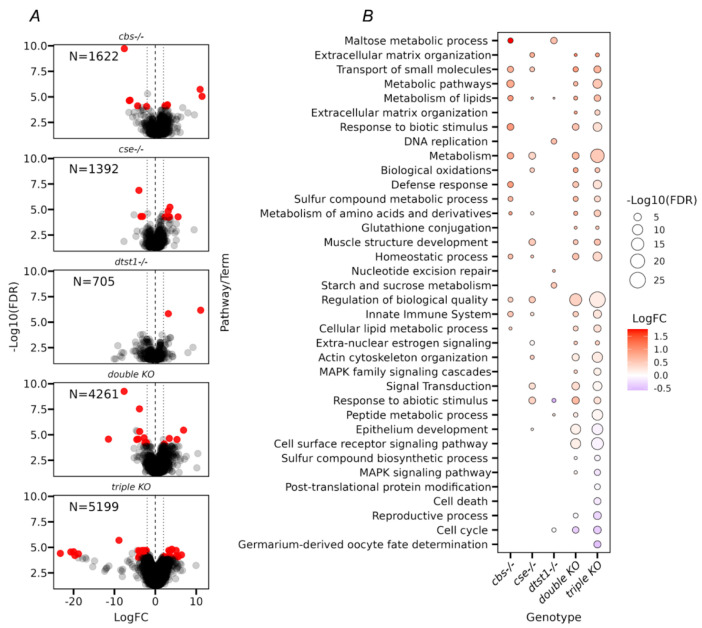
Transcriptomic analysis of gene expression in adult female flies. (**A**) Volcano plots with numerical values of differentially expressed genes (DEGs) in the top left side of each plot (FDR < 0.05). (**B**) GSEA (gene set enrichment analysis) of the DEGs (FDR < 0.05) in whole fly tissues was performed using ShinyGO v.77 software. The X-axis represents different KO strains; the Y-axis represents enriched pathways from the Gene Ontology database “Biological Process” category. The size of each bubble represents the significance of enrichment (−log10 false discovery rate) according to the Fisher test. All bubbles in the figure indicate that the result passed the FDR < 0.05 significance cutoff. The color of each bubble indicates the averaged Log2FC value of all the genes included in the category.

The volcano plots in Figure 3A show that the greatest number of changes were found in the double- and triple-KO strains, which had a tendency to upregulate gene expression. The comparative analysis of Gene Ontology terms revealed multiple metabolic changes in the studied strains (Figure 3B). Thus, activation of several body defense systems (e.g., defense response, response to biotic and abiotic stimuli, and activation of the immune system) was observed (Figure 3B). The observed pattern of DEGs indicated the presence of chronic stress in the double and triple KOs, which may lead to reduced lifespans [50].

It is also evident from Figure S3 that double and triple KOs with reduced H_2_S production were susceptible to oxidative stress, as indicated by the induction of transcription of genes belonging to the CYP (Cytochrome P450), GST (glutathione S-transferase), and UGT (uridine diphosphate-glucuronosyltransferase) families. It is of note that changes in the transcription of genes responsible for many metabolic pathways were also observed (Figure S4). It is known that metabolism plays an important role in the regulation of aging through different mechanisms. Metabolic reprogramming leads to impaired organismal vitality, age-dependent increases in disease susceptibility, and a decreased stress response capacity, and represents one of the major driving forces of aging [51]. Interestingly, the GO analysis of the *dtst1-/-* strain revealed increased expression of genes responsible for DNA replication and repair (Figure 3B and Figure S5) and sucrose and maltose metabolism, which may explain the increased longevity described for this strain [52,53,54]. The moderate reduction in hydrogen sulfide levels in this strain probably leads to a reduction in oxidative stress, which also may increase the lifespan (Figure 1 and Figure 2).

Acute hydrogen sulfide deficiency in the double and triple-knockout strains can lead to chronic oxidative stress, which may affect mitochondrial function. Hydrogen sulfide plays central roles in mitochondrial homeostasis and function, and it is known that both an excess and deficiency of H_2_S may have deleterious effects at the cellular and organism levels [55,56,57]. We analyzed the transcription of genes related to mitochondrial function in the KO strains (e.g., Krebs cycle enzymes, respiratory chain components, ATP synthase, etc.).

**Figure 4:**
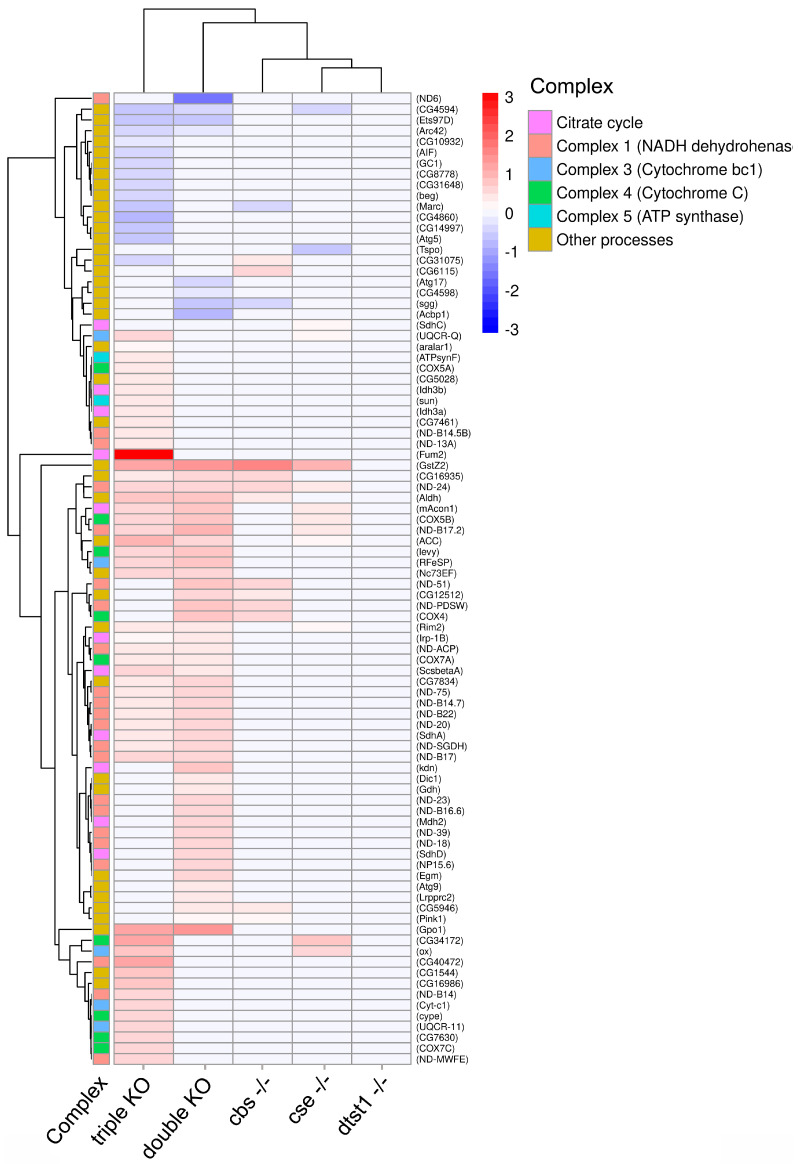
Heat map showing the expression level of genes involved in redox processes in mitochondria in KO strains compared to control flies. Pairwise comparisons with control strain are shown in red (positive log fold-change (log2FC), FDR < 0.05) and blue (negative log2FC, FDR < 0.05).

As shown in Figure 4, KO of the *dtst1* gene did not result in any DEGs compared to the control strain. Similarly, single deletions of the *cbs* and *cse* genes caused only minor changes in the transcription of these genes, while there were differences between these strains. In contrast, the double and triple KOs led to dramatic changes in the transcription of genes involved in mitochondrial function. Surprisingly, the triple KO exhibited a different transcriptional landscape compared to the double-knockout strain (Figure 4). Thus, knockout of the *dtst1* gene in the background of a double knockout showed effects that were absent in the case of a single knockout of *dtst1*, i.e., the presence of intact *cbs* and *cse* genes completely compensated for the impact of the *dtst1* gene knockout.

The double and, in particular, triple KOs of the studied genes resulted in oxidative stress and compensatory upregulation of specific mitochondrial genes (NADH, cytochromes, and ATP synthase). This apparently adaptive response represents an effort to counteract metabolic stress, restore redox balance, and meet the heightened energy demands. Changes in the intensity of oxidative phosphorylation are associated with changes in lifespan. In particular, increased oxidative processes in mitochondria can lead to oxidative stress and mitochondrial DNA damage involving ROS, which may result in accelerated aging [58]. More significant changes in the expression of genes encoding the components of the respiratory chain complexes were found in the double and triple knockouts (Figure 4), which correlated with the observed decreased longevity.

### 3.3. Fecundity Changes in the Knockout Strains

**Figure 5:**
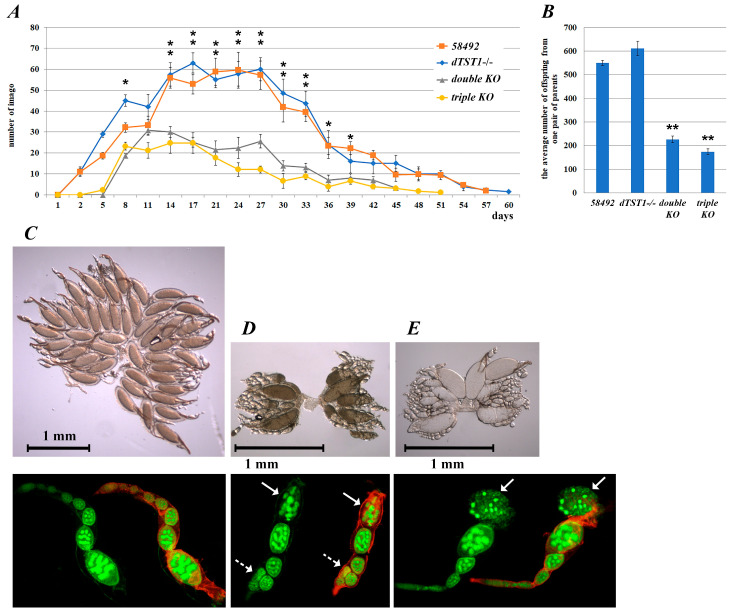
Fecundity analysis of studied strains. (**A**) Dynamics of reproductive capacity of one pair of individuals during life. Eclosed flies were counted every three days (after second day). Significance (* *p* < 0.05, ** *p* < 0.01) was determined by two-way ANOVA followed by post hoc Tukey’s HSD tests. (**B**) Average number of offspring from one pair of parents for whole reproductive period. Results of three experiments were taken into account. Statistical significance was calculated using Student’s *t* test. (**C**–**E**) Ovaries and individual ovarioles from 4-day-old female control (58492), double-, and triple-KO flies. Dashed arrows indicate spatial structure disorder and solid arrows indicate oocyte death in ovarioles of double- and triple-KO strains. Immunofluorescent DNA staining of ovarioles was performed using SYTOX Green (nucleic acid stain) and Phalloidin-Atto Rho6G antibody (F-actin stain, red).

Since fecundity is an important indicator of *Drosophila* females’ viability, we investigated the effects of the knockouts on fecundity parameters and monitored the duration of the effective reproductive period in all the KO strains in comparison with the control flies. The analyses showed that KO of *dtst1* increased the average fecundity by 13% (Figure 5A,B) while the duration of the reproductive period in this strain was not changed compared to the control strain (Figure 5A; Table S3A). On the other hand, the duration of the reproductive period in the females from the double- and triple-KO strains decreased by 16% and 11% (*p* < 0.05), respectively, compared to the control strain (Figure 5A; Table S3A). Interestingly, the double-KO flies exhibited a 59% decrease in the number of offspring from a single pair of parents, while the triple-KO flies demonstrated a 68% decrease in this parameter (Figure 5A,B; Table S3B). The reduced number of progeny observed in the knockout strains appeared to be caused by abnormalities in ovarian and individual ovariole development (see Figure 5C–E).

Figure 5C–E shows that the flies from the control strain had normally developed ovaries with normal ovarioles. In contrast, the double-KO females exhibited a strong delay in ovarian development. We observed a significant number of dead ovarioles (up to 5%) in the oviductal chambers at the eighth stage of development and disturbances in the spatial orientation of the oviductal chambers at the first and third stages of development in the KO flies. The multiple oogenesis defects in the KO females require further detailed analysis.

To further characterize the fecundity patterns [59] of the strains, a comparative analysis of the relationship between the age of the parents and the percentage of offspring (fecundity rate) was performed. The fecundity rate was quantified as the percentage of offspring produced every 3 days relative to the total number of offspring (Figure S6).

The highest fecundity rate was observed in eight- to seventeen-day-old flies from the triple-KO strain and eleven- to fourteen-day-old flies from the double-KO strain. Interestingly, the double and triple-KO strains exhibited significantly higher fecundity rates than the control and *dtst1-/-* strains at these time points. In the triple and double-KO flies, the fecundity began to decline on the fourteenth–seventeenth day after eclosion, whereas in the control and *dtst1-/-* strains, it reached a plateau on the fourteenth day and did not decline until the 27th day after eclosion (Figure S6). Thus, the progeny of the double- and triple-KO strains were predominantly produced from relatively young parents, which correlates with the observed earlier senescence of these strains (Figure 2).

**Figure 6:**
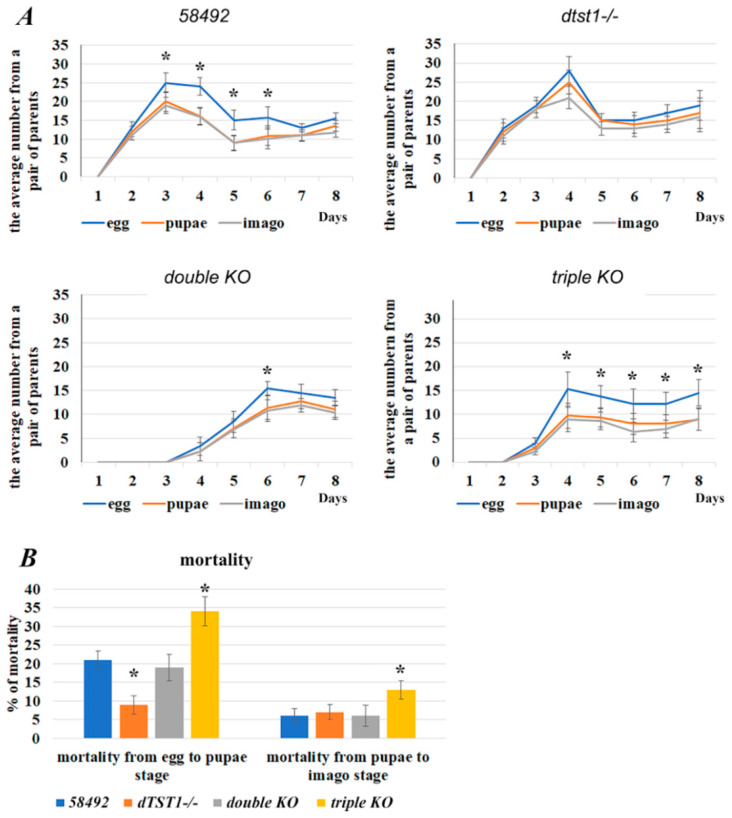
The number of progeny and mortality levels at different stages of ontogenesis of the studied strains. (**A**) The daily average number of eggs, pupae, and adults from one female over eight days. (**B**) Percent mortality of tested strains at embryonic, larval (left), and pupal stages (right). * *p* < 0.05. The results of three experiments were taken into account. Statistical significance was calculated using Student’s *t* test.

To estimate the effect of the knockouts on the different developmental stages of *Drosophila*, the number of eggs laid by one pair of flies was counted, followed by pupa and adult counts (Figure 6A).

The mortality of the flies at the embryonic and larval stages was also determined as the ratio of the number of pupae to the total number of eggs laid (Figure 6B). The mortality at the pupal stage was determined as the ratio of the number of eclosed adults to the number of pupae (Figure 6B).

The lowest mortality at the embryo/larvae stages was observed in the *dtst1-/-* strain, while the highest was in the triple-KO strain (Figure 6). Similarly, mortality during metamorphosis at the pupae stage was highest (13%) for the triple-KO flies compared to the control strain (Table S3C). Thus, surprisingly, KO of the *dtst1* gene improved several fecundity parameters. Our transcriptomic data revealed that the single deletion of *dtst1* can affect DNA replication and repair processes (Figure S5), which can have positive effects at different developmental stages. However, in the case of the triple KO, we observed a significant increase in developmental mortality and a reduced reproductive capacity in comparison with the double-KO and control flies.

### 3.4. Transcriptome Analysis of Ovaries from the Studied KO Strains

**Figure 7:**
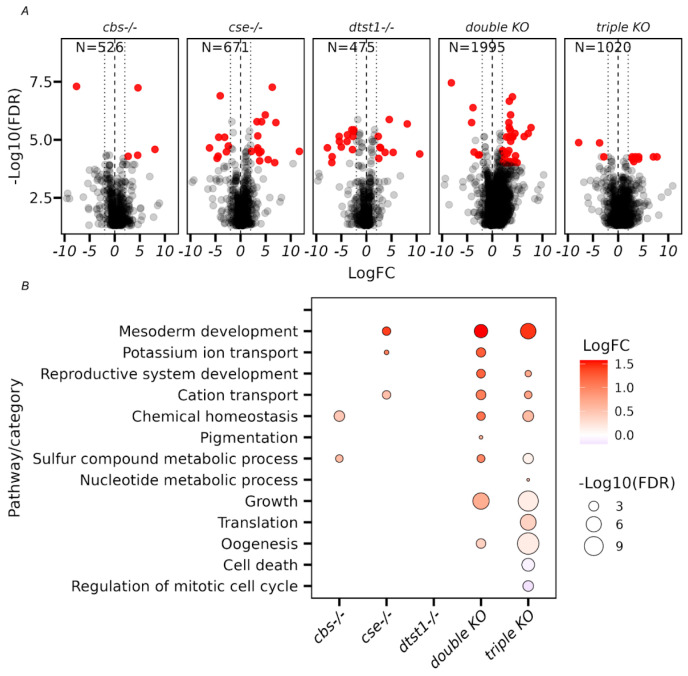
Transcriptome analysis of the ovaries of the studied strains. (**A**) Volcano plots. Vertical dotted lines representing −2 and 2 values of LogFC (**B**) The GSEA results for differentially expressed genes (FDR < 0.05) in ovaries. The X-axis represents the different KO strains, and the Y-axis represents the enriched pathways from the Gene Ontology database “Biological Process” category. The size of each bubble indicates the significance of enrichment (−log10 false discovery rate), according to the Fisher test. All bubbles presented in this figure passed the FDR < 0.05 significance cutoff. The color of each bubble indicates the average Log2FC value for all the genes included in the category.

To identify possible reasons for the observed inter-strain differences in the various reproductive parameters (Figure 5 and Figure 6), we performed an analysis of the transcriptome changes in the ovaries of all the studied strains. The transcriptome analysis allowed us to conclude that in the case of isolated ovaries, the double- and triple-KO strains had significantly more genes with altered transcriptional activity compared to the strains containing knockouts of single genes (i.e., *cbs*, *cse*, and *dtst1*) (Figure 7A).

Interestingly, the analysis of common DEGs in the ovaries of all the studied strains with single knockouts (*cbs-/-* and *cse-/-*), as well as the double- and triple-KO strains revealed enrichments in GO categories such as mesoderm development and muscle cell differentiation (i.e., myofibril assembly, sarcomere organization, and actin cytoskeleton organization) (Figure 7 and Figure S7). Thus, even a single deletion of the *cbs* or *cse* gene affected muscle formation and other features of morphogenesis.

On the other hand, the deletion of *dtst1* did not lead to an enrichment of these categories. Its deletion mainly led to the enrichment of categories related to the metabolism of organic cyclic compounds (Table S4), which can influence oocyte growth, maturation, and developmental competence.

**Figure 8:**
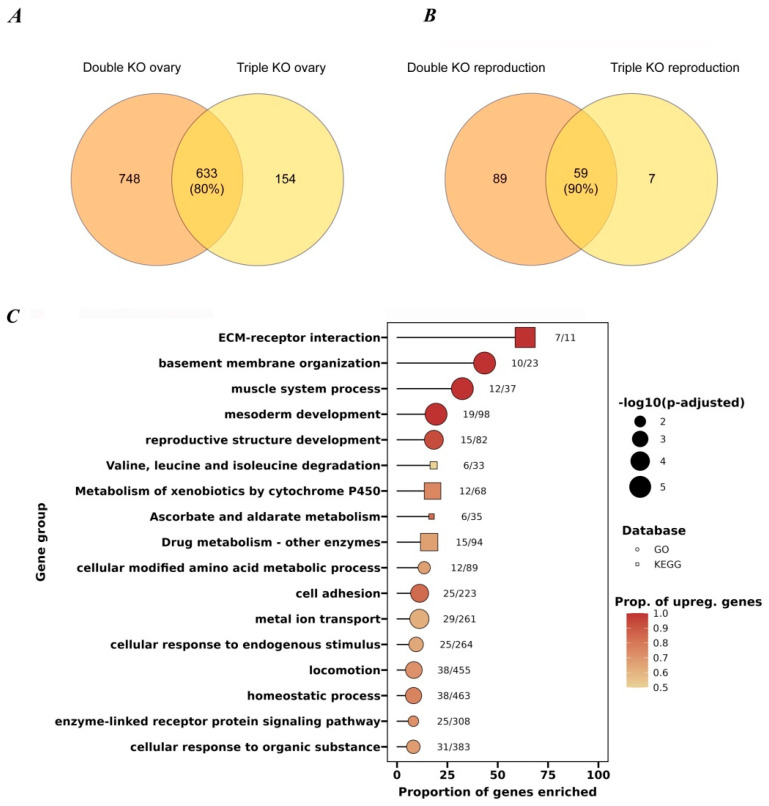
Venn diagram and GSEA (gene set enrichment analysis) results for DEGs in ovaries of double- and triple-KO strains. (**A**) Overlapping DEGs in ovaries of double and triple KOs (80% in common). (**B**) Overlapping DEGs involved in reproductive function in triple and double KOs (90% in common). (**C**) GSEA results for the most enriched terms for common differentially expressed genes in ovarian tissue in double and triple knockouts. The X-axis shows the ratio between the number of enriched genes and the total number of genes in each term. The Gene Ontology terms are shown as circles and KEGG pathways are shown as squares. The color of each figure reflects the proportion of upregulated genes in each term.

To verify that it was the KOs of *cbs* and *cse* that were responsible for most of the abnormalities observed in the ovaries of the double and triple knockouts, we identified DEGs common to both strains (Figure 8A). The figure shows that the triple knockout shared 80% of the DEGs of the double knockout. Moreover, a comparison of reproduction-related genes based on the GO data showed a 90 percent overlap in DEGs between the triple and double knockouts (Figure 8B).

The observed functional enrichment of the common DEGs in the double and triple knockouts suggests that these genes form important interrelated groups. These groups with upregulated DEGs included genes involved in muscle system processes, mesoderm development, basement membrane organization, extracellular matrix (ECM) organization, and reproductive structure development (Figure 8C; Tables S5 and S6). Earlier, it was shown that even single ECM components contribute to the mechanical properties of the basement membrane, controlling tissue and organ shape [60]. Therefore, the observed changes in the expression level of these gene groups may be responsible for the observed abnormalities in ovarian development and reproduction of the KO flies. Moreover, in the transcriptomes of both the ovaries and whole flies, we found an increase in the level of transcription of genes belonging to families such as the CYP, GST, and UGT families (Figure S3), which indicates that the need to eliminate toxic substances can negatively affect the processes of oogenesis.

### 3.5. Changes in Malpighian Tubule Structure and Function in Double- and Triple-Knockout Strains

It can be concluded from the above that deletions of genes involved in H_2_S synthesis and metabolism affect, to varying degrees, many vital processes and life-history traits in *Drosophila* flies, such as lifespan and fecundity. The transcriptome analysis revealed the upregulation of genes belonging to families such as the CYP, GST, and UGT families that play critical roles in detoxification, stress responses, and physiological processes.

It is known that CYP enzymes oxidize xenobiotics, GST conjugates glutathione to electrophilic toxins, and UGT catalyzes glucuronidation (adding glucuronic acid) to lipophilic substrates. After the detoxification of toxins through oxidation, conjugation, or glucuronidation, the modified metabolites must be excreted to prevent cellular damage. Previously [26], a transcriptomic analysis of the double-KO strain showed a significant increase in the expression of several genes controlling the fly excretory system (e.g., *capa*, *capaR*, *Uro*, etc.) and genes related to potassium channel function (*Irk3*, *irk2*, and *KCNQ*). All these genes play important roles in the function of the *Drosophila* excretory system. The observed increase in their expression probably indicates a disruption of fluid homeostasis and the urgent need for active excretion of toxins and other harmful substances from the organism.

In recent years, the functions of the Malpighian tubules (MTs) of *Drosophila* and other insects have attracted much attention [61,62,63,64]. This organ, which corresponds to the kidneys of mammals [61,63,65], plays a critical role in the normal metabolism of flies and other insects. Since we have previously described [26] the increased expression of several genes involved in excretion processes in the KO strains, we decided to analyze the MT structure in these strains. The MTs were stained with the fluorescent dye SF7 to detect differences in the production of H_2_S. The experiments showed that the MTs of all the strains produced H_2_S (Figure S8).

**Figure 9:**
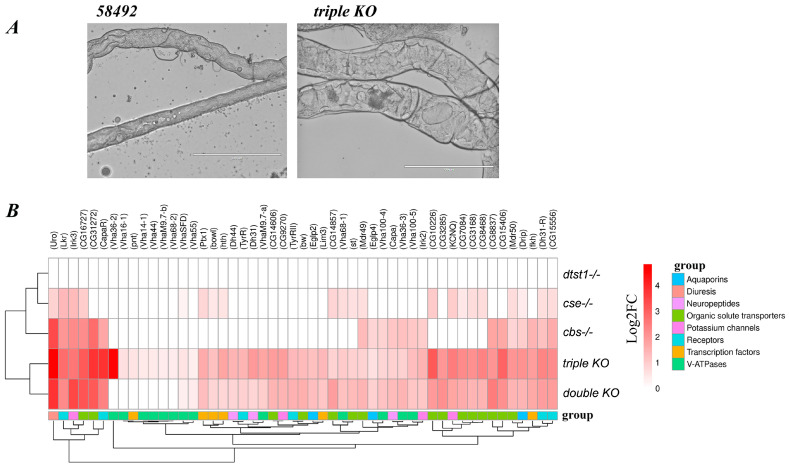
The structure of the MTs and the expression of pertinent genes in the control and KO strains. (**A**) Photographs of the main segment of MTs from the control and triple KO at the same magnification (10×) were taken using an Evos FL microscope (scale: 200 μm). (**B**) Heat map shows the expression of genes related to MT function in females of the KO strains compared to the control strain 58492. Red indicates a positive log fold-change (log2FC; FDR < 0.05).

The study of MT morphology and size allowed us to conclude that in the *dtst1-/-* KO strain, the MTs did not differ from those of the control strain. They were thin and long, varying from 25 to 40 µm in thickness and up to 3000 µm in length. In the control strain, the thickness of the MTs varied from 30 to 50 μm, and the length varied from 2500 to 3000 μm. Minor vessel thickening of the MTs was observed in the *cse-/-* and *cbs-/-* (KO) strains, while in the double-KO flies, the thickness of the MTs reached 45–75 μm. The analysis showed that the MTs of the triple-KO strain exhibited the largest changes in size and structure (Figure 9A and Figure S8). Thus, the MT thickness in the triple-KO flies reached up to 120 μm, while the total length never exceeded 2000 μm. Next, we investigated the expression of genes involved in the functioning of MTs by analyzing the transcriptome libraries generated from five-day-old females (Figure 9B). In our comparative analysis of the transcriptome, we explored the previously described functional transcriptome of *D. melanogaster* MTs [66]. It was shown that the KO of the *dtst1* gene does not affect gene expression in the MTs (Figure 9B). At the same time, the combination of this KO with deletions of the *cbs* and *cse* genes (triple KO) resulted in a drastically altered MT morphology (Figure 9A) and increased expression levels of the genes involved in MTs’ physiological activity (Figure 9B).

Thus, Figure 9 shows that in all the KO strains (except *dtst1-/-*), the expression level of the *uro* gene, which encodes an enzyme localized in MTs and involved in the oxidation of uric acid to allantoin, was dramatically increased.

**Figure 10:**
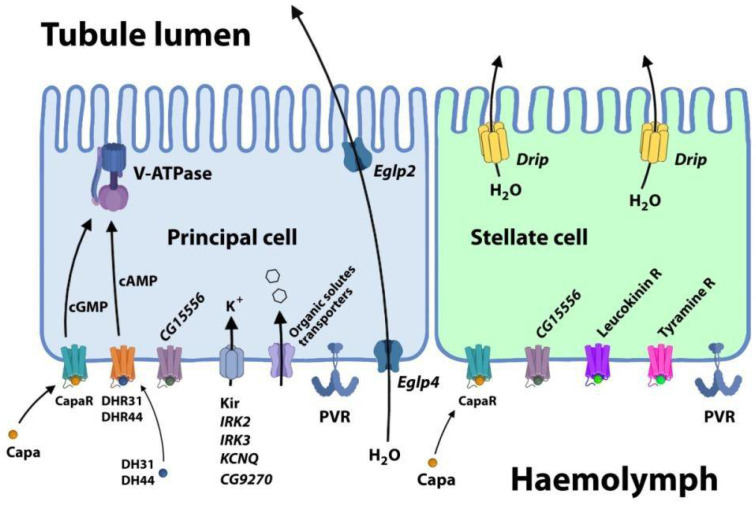
Overview of products of genes with increased expression in the double- and triple-KO strains that function in principal and stellate cells and play important roles in the physiology of MTs: V-ATPase, a proton pump that provides the driving force for transepithelial ion and fluid secretion in insect Malpighian tubules (MTs); Eglp2/4 and Drip transport water across the cell membrane; Capa and CapaR stimulate fluid secretion in Malpighian tubules; DH31/DHR31/DHR44 are diuretic hormones/receptors; and Kir inwardly rectifying K^+^ channels (IRK2/3, KCNQ, and CG9270) support the flow of positively charged K^+^ ions into the cell, pushing the membrane potential back to the resting potential. Organic solute transporters include the following subgroups: monocarboxylate transporter—*CG8028* and *CG8468*; sugar transporters—*CG15406*, *CG31272*, *CG8837*, and *CG3285*; ABC transporters—*CG9270*, *brown*, *CG10226*, *scarlet*, *mdr50*, and *mdr49*; and organic cation transporters—*CG3168*, *CG14857*, *CG7084*, and CG16727. These transporters are involved in the transport of small organic molecules such as metabolites, signaling molecules, antioxidants, and xenobiotics. PVR—a receptor tyrosine kinase; *CG15556*—a G-protein-coupled receptor; and leucokinin and tyramine receptors—G-protein-coupled receptors activated by leucokinin or tyramine. These receptors transmit signals via intracellular calcium and are involved in the regulation of fluid secretion levels.

A pronounced increase in the expression of several groups of genes related to metabolism and excretion was observed in the double KO and especially the triple KO. These genes encode receptors localized on the surface of principal cells, i.e., CapaR, DHR31, and peptides that bind to them (CAPA, DH31, and DH44, which act as diuretic hormones). Diuretic hormones (DH31 and DH44) are known to stimulate fluid secretion in MTs through the activation of V-ATPases in the principal cells of the main segment of MTs [61,67]. The expression of V-ATPases was increased in *Drosophila* females with the triple KO and, to a lesser extent, those with the double KO (Figure 9B and Figure 10). The expression of genes that encode receptors on the surface of stellate cells, such as leucokinin and tyramine receptors, was also upregulated in the KO flies. Similarly, the expression levels of several transcription factors that are enriched in MTs, i.e., *pnt*, *ptx*, *bowl*, *hth, fkh*, *Ets21C*, *Lim3*, were significantly elevated in the double- and triple-KO flies (Figure 9B).

The expression levels of genes belonging to the superfamily of internal rectifier potassium channels (Kir), which play an important role in hindgut and MT osmoregulation [68], were also altered in the KO strains (Figure 9B).

In all the KO strains except *dtst1-/-*, the expression level of the *drip* gene, which is involved in water transport through the cell membrane, was significantly increased. The expression levels of the *eglp4* and *eglp2* genes, which are involved in the function of water channels, were also increased in the double- and triple-KO strains (Figure 9B). It is evident from Figure 9B that the expression levels of organic solvent transporters were increased in the strains with a deletion of the *cbs* or *cse* gene. Similarly, genes encoding monocarboxylate transporters such as *CG8028* and *CG8468* showed increased expression in the strain with the *cse* gene deletion, while genes encoding sugar transporters, i.e., *CG15406*, *CG31272*, *CG8837*, *CG3285*, and *CG14606*, exhibited increased expression in the strain with the *cbs* gene deletion. This could be attributed to the accumulation of different metabolites in these strains, i.e., an increase in methionine and toxic homocysteine levels in the *cbs-/-* strain and an increase in cystathionine levels in the *cse-/-* strain [23]. It is of note that the expression of all these genes was significantly increased in the triple- and double-KO strains (Figure 9B). This gene is essential for female reproductive functions

## 4. Discussion

The high conservation of the ancient adaptogenic system responsible for the synthesis of one of the three major gas transmitters (H_2_S) allows us to use *Drosophila* flies as a reliable and convenient model for studying the role of this gas in virtually all processes associated with life-history traits, including reproduction, aging, immune responses, and even mating behavior. One convenience of this model is the possibility of performing comparative analyses of the effects of individual gene knockouts and combined (double and triple) knockouts on the transcriptome and life-history traits, which is important for understanding the contribution of genes to various pathological processes in humans. Previously, we generated KOs of two major TSP genes (*cbs* and *cse),* as well as a third gene (*CG12279*) using CRISPR/Cas9 technology. We also analyzed the effect of deletions of these genes on transcription and several vital parameters in flies [23,24,25]. In the present study, we continued this investigation by generating a strain with KOs of all three genes, including a third gene (*CG12279*), which, like *3-mst*, belongs to the thiosulfate sulfurtransferase family (TST). The bioinformatics analysis demonstrated that this gene encodes a protein with a single rhodanese domain. Proteins containing rhodanese domains belong to a widespread protein family involved in sulfur transfer reactions between various donor and acceptor molecules and catabolism of H_2_S [32,69,70]. Importantly, in our case, the studied gene (*CG12279*) encodes a protein with an unusual active site. This site contains a catalytic cysteine followed by a positively charged lysine and a hydrophobic alanine, whereas the catalytic cysteine of canonical TST is followed by two charged amino acids [33] (Figure S9). Notably, the programs MitoFates [71], TPpred3 [72], and TargetP 2.0 [73] predict that the protein encoded by the *CG12279* gene should localize in the cytoplasm and not in the mitochondria, which is a characteristic feature of canonical TST family enzymes. TST and MST have similar physicochemical and catalytic properties because they are evolutionarily related enzymes belonging to the same family [31,32,74,75]. The catalytic activity of these two enzymes depends on the cysteine residue in their active site (reviewed in [76]). It was shown that in mice with knockout of the *3-mst* gene, the expression of the *tst* gene is increased as a compensatory mechanism [74].

Since the nucleotide sequence analysis showed that *CG12279* is structurally homologous to human *tstd1,* we designated it as *Drosophila tst1* or “*dtst1*”. It is necessary to emphasize that phylogenetic analysis has shown that arthropods, including *Drosophila*, do not have the canonical *3-mst* gene in their genome [27]. It should be mentioned that bioinformatics allowed us to detect three genes with a single rhodanese domain in the *D. melanogaster* genome. Two of them are highly homologous (*CG12279* and *CG4456*) and encode polypeptides that are 111 amino acid residues in length. Because the *CG12279* gene, which we named *dtst1*, is expressed at all developmental stages, including adult flies, we chose it for further analysis. Interestingly, the sequence of another gene in this family (*CG4456*) in the *Drosophila* genome overlaps with the sequence of the *hsp22* gene, and according to the Flybase data, this gene is also stress-inducible and has a similar expression pattern to *hsp22*. The third gene in this family (*CG6000*) differs from *CG12279* and *CG4456*. It has a different intron–exon structure and encodes a protein that is 154 amino acids long. Interestingly, our transcriptomic data showed that in triple-KO flies, *dtst1* KO causes a compensatory increase in the expression level of the *CG6000* gene, but not of the stress-inducible gene *CG4456* (Figure S10). Thus, we inactivated a sulfurtransferase gene encoding a protein with a single rhodanese domain that appears to be involved in transferring sulfur from protein to protein and is involved in cyanide detoxification. We studied the role of dTST1 at the cellular and organismal levels, both separately and in complex with the major TSP genes (*cbs* and *cse*) involved in H_2_S synthesis.

Deletion of the *dtst1* gene significantly reduced, the production of H_2_S in the fly body, although it was still at a fairly high level. This suggests that dTST1 is most likely not engaged in the degradation of H_2_S in mitochondria [34] but is probably involved in sulfur transfer processes [33]. dTST1 may transfer sulfur from a donor (e.g., thiosulfate) to form an intermediate persulfide, which can be reduced by thioredoxin to H_2_S, similar to the mechanism of 3-MST [77]. The level of H_2_S production in the double and triple knockouts was very low and difficult to accurately quantify (Figure 1). The obtained results suggest that H_2_S in flies is predominantly synthesized by canonical enzymatic pathways, and the contribution of non-canonical and non-enzymatic pathways to its production is not significant.

The knockout of *dtst1* had an unexpected effect on various fly life-history traits and metabolism. The deletion of this gene alone increased fecundity and caused a significant increase in the lifespan of females (Figure 2 and Figure 5). At the same time, our previous transcriptomic analysis [26], as well as the present studies, showed that knockout of the *dtst1* gene only has a very minor effect on gene expression in the fly body and ovaries (Figure 3 and Figure 7). However, the enrichment analysis of KEGG pathways identified two groups of DEGs that could be responsible for the observed increase in lifespan and reproduction in the *dtst1* knockout. The genes exhibiting increased expression in the *dtst1* KO flies are associated with DNA replication, mismatch repair, and sucrose metabolism (Figure 3B and Figure S5) [78,79,80]. A recent study [81] showed that endogenously produced cyanide in mammalian cells and tissues performs a variety of physiological functions, including support of mitochondrial respiration, stimulation of cell proliferation, and cytoprotection. We speculate that the positive effects of deleting the *dtst1* gene on several aspects of *Drosophila* physiology, such as longevity and reproduction, may be due to increased levels of cyanide, which, at physiological concentrations, provides cytoprotective effects and increases cell proliferative activity. However, this hypothesis requires further research. In our study, we found that deletion of key genes involved in the transsulfuration pathway (TSP) and H_2_S metabolism (*cbs*, *cse*, and *dtst1*) significantly affects longevity, fertility, as well as the mitochondrial and excretory functions of the flies. It is well documented that deletion of the *cbs* and *cse* genes decreases the production of H_2_S as well as the levels of cysteine [82,83]. Low levels of cysteine result in a decrease in glutathione (GSH) synthesis and, hence, result in oxidative stress [84]. Besides increasing the methionine concentration [23,83], the deletion of these genes probably increases the level of S-adenosylmethionine (SAM), which is the principal donor for histone methylation. Therefore, the disruption of methionine metabolism can directly affect histone methylation levels and influence multiple biological processes, including longevity and reproduction parameters [85,86]. On the other hand, methionine itself can act as an antioxidant and is one of the main targets for reactive oxygen species (ROS). It is known that methionine residues on the surface of native proteins can be oxidized by ROS to R- and S-methionine sulfoxide, which can be reduced to methionine by methionine sulfoxide reductases (*msr*). Consistent with this, a significant upregulation of *msrA* in the *cbs-/-*, double, and triple KOs was observed (Figure S4). This process is involved in cell signaling and can target proteins for proteolytic degradation [87].

The oxidative stress induced by the double deletion of the *cbs* and *cse* genes leads to activation of the *CncC* gene (*NRf2* orthologue), whose activity is modulated by the redox state of the cell; it regulates the expression of genes involved in antioxidant defenses [88,89]. It is of note that in *Drosophila*, CncC also plays an important role in stem cell proliferation and in the neuroendocrine axis that coordinates insect metamorphosis [90,91]. Chronic activation of this gene can affect these processes.

Interestingly, the transcriptome analysis of strains with the *cbs* gene deletion, and especially with the double- and triple-gene deletion (Figure S3), revealed increased expression of genes containing antioxidant response elements (AREs) for CncC binding. Deletions of the *cse* and *dtst* genes had a weaker effect on this process. Genes with increased expression included GSTs, UGTs, and CYPs. It is known that GSTs conjugate glutathione (GSH) to xenobiotics, electrophiles, and lipid peroxidation byproducts, neutralizing their toxicity and facilitating excretion. GSTs may also reduce oxidative damage by quenching reactive oxygen species (ROS) and recycling oxidized glutathione (GSSG) back to GSH. Certain GST isoforms regulate various important signaling pathways (e.g., the JNK and apoptosis pathways) [92,93]. The main function of UGTs is to conjugate glucuronic acid to xenobiotics (e.g., plant allelochemicals and pesticides) and endogenous compounds such as hormones and neurotransmitters to enhance the excretion process [94,95]. The demonstrated upregulation of GSTs and UGTs in *cbs-/-* and especially in the double and triple KOs represents an adaptation to the oxidative stress and toxin overload occurring in these strains [96]. Moreover, the accumulation of homocysteine, an intermediate product of the TSP, in the body of strains with a *cbs* gene deletion should cause additional intoxication and oxidative stress [97,98]. An amino acid analysis revealed an 8-fold increase in methionine/homocysteine levels in the *cbs-/-* and double-knockout strains and a 170-fold increase in cystathionine levels in the *cse-/-* strain [23]. The accumulation of the toxins and products that are neutralized by GSTs and UGTs results in the activation of the excretory system, which was observed in the KO flies. We observed both morphological changes in the MTs and an increase in the expression of several groups of genes related to metabolism and excretion in the double-KO and especially triple-KO flies (Figure 9). It is known that MTs, like the kidneys in mammals, purify the hemolymph by removing waste metabolites and toxins [99]. In addition, it is known that in mammals, H_2_S plays important roles in renal physiology and pathogenesis [100].

Intoxication in the strains with the *cbs* gene deletion, as well as in the double and triple-KO flies, was evidenced by an increase in the expression level of the DDT resistance gene *cyp6g1* (Figure S3), which has previously been shown to be overexpressed in the MTs compared to the entire fly body [101,102]. The increased expression level of ABC-type transporter family genes (*mdr50* and *mdr49*) (Figure 9), which confer multidrug resistance [103,104], was observed in the knockout strains, which also indicates intoxication in these flies. In this study, the most pronounced changes in MT morphology and transcriptomic alterations were observed in the triple knockout. We suggest that the increase in cyanide levels in the background of the deletion of the *cbs* and *cse* genes leads to homocysteine accumulation and oxidative stress, increasing the intoxication of the organism.

Energy expenditure is required to activate the body’s defense systems and eliminate toxins in the KO flies. Moreover, H_2_S deficiency probably reduces the scavenging of reactive oxygen species (ROS), increasing mitochondrial oxidative stress. These factors should affect mitochondrial function, and we indeed observed increased expression levels of several mitochondrial genes in the double- and triple-KO flies (Figure 4). Certain differences between the double and triple KOs are probably due to the influence of the *dtst1* gene on mitochondrial function (possibly due to the slight increase in cyanide levels), which is inconspicuous in the presence of intact *cbs* and *cse* genes. However, knockout of the *dtst1* gene in the background of a double knockout of the *cbs* and *cse* genes enhances the effects, which indicates a complex interaction between these genes. Undoubtedly, metabolome analysis is necessary to confirm the role of the *cbs*, *cse*, and *dtst1* genes in all the studied processes.

The reduced fecundity observed in the double- and triple-knockout flies was due to the observed abnormalities in ovarian development and impaired oogenesis. The oxidative stress present in the KO flies likely caused changes to the endocrine system, which led to a redistribution of energy from reproduction to survival [105,106]. It has been shown that *D. melanogaster* females respond to thermal and nutrient stressors by arresting egg production due to the elevated level of the steroid hormone ecdysone. Recent research in *Drosophila* has unveiled key aspects of the role of ecdysone in insect oogenesis [107,108,109]. Elevation of steroid levels results in arrested oogenesis and reduced octopaminergic input to the reproductive tract, suppressing ovulation [109]. Notably, the expression of several genes involved in ecdysteroid biosynthesis, namely *Cyp4aa1*, *Cyp311a1*, and “*shadow*”, was upregulated in both the double and triple knockouts (Figure S3). We also found an increase in the expression level of ecdysteroid kinase-like family genes that act on small-molecule substrates and may function in detoxification processes, especially in the double- and triple-KO strains (Figure S11). To confirm this hypothesis, further analyses of ecdysone levels in these strains should be performed.

It is known that the highest level of *cbs* and *cse* expression is in *Drosophila* ovaries (https://flybase.org/reports/FBlc0003498.html (accessed on 15 February 2025)). Similarly, in mice, *cbs* is also strongly expressed in follicular cells at different stages of development, and studies of *cbs*-deficient mice demonstrated that this gene is essential for female reproductive functions [110]. It has also been shown in mammals that the endogenous hydrogen sulfide production system (*cbs* and *cse*) regulates ovulation and plays an important role in multiple processes associated with female reproduction [21].

Our data demonstrate that deletions of the *cbs* and *cse* genes result in increased expression levels of genes associated with reproductive structure development (ECM–receptor interactions, basement membrane (BM) development, and muscle development) (Figure 7, Figure 8 and Figure S7; Table S6), which may cause ovarian fibrosis. Ovarian fibrosis results in ovarian dysfunction and is characterized by excessive proliferation of ovarian fibroblasts and the accumulation of extracellular matrix (ECM) [111].

It is known that the ECM supports epithelial tissues and is composed of highly conserved components, including collagen IV (Col IV) and Laminin [112]. These components are important for a number of morphogenetic events that occur during oogenesis, including cell migration, proliferation, elongation, and intercalation.

There is also evidence that the BM represents an active signaling platform that is involved in many developmental processes, such as cell differentiation, proliferation, survival, polarization, and cell migration [112,113,114,115]. Thus, the changes in expression levels of components of these pathways that were observed in the knockout flies may lead to various abnormalities in ovarian development and function. In the KO strains, an imbalance in many physiological programs involved in the normal physiological activity of *Drosophila* took place. Our comprehensive study showed that in *Drosophila*, as well as in humans and other organisms, the genes responsible for the synthesis and metabolism of H_2_S are involved in a wide spectrum of life-history traits.

It has been demonstrated in the last decade that H_2_S synthesis disturbances contribute to different human diseases, including cardiovascular diseases, viral infections, neurological disorders, reproductive pathologies, and various hyperhomocysteinemia-induced dysfunctions [110,116,117]. However, the molecular mechanisms through which H_2_S contributes to different diseases are complex and require convenient models for analysis. *Drosophila* flies containing single and double KOs of major genes involved in H_2_S production represent a unique model for investigating the role of this gas and the TSP in the life-history traits and functioning of mitochondria and the reproductive and excretory systems.

## 5. Limitations

No confirmation of the knockout of the studied genes at the protein level was performed. All the experiments reported here were performed using only females of the KO strains. Experiments on males from these KO strains will be performed in due time. The transcriptomic analysis was performed either on RNA from ovaries or on RNA from whole females. Therefore, tissue heterogeneity in the samples may offset changes in the expression levels of tissue-specific genes. Similarly, the H_2_S production measurements were performed using whole bodies with intact intestines, but not individual organs (e.g., MTs, ovaries, etc.), which may introduce additional uncertainty into the measurements and conclusions. The main goal of this study was to determine the effect of deletion of individual genes involved in sulfur metabolism, as well as their combined (double and triple) knockouts on gene expression and the physiology of *Drosophila*; metabolomic studies of the individual knockout strains have not yet been performed.

## 6. Conclusions

Our studies using strains with KO of the major TSP genes, as well as the gene described by us encoding a single-domain rhodanese (*dtst1*) that is involved in sulfur transfer, demonstrated that this gas transmitter is involved in many physiological processes in the fly. It was also shown that while knockout of *dtst1* only slightly reduced H_2_S levels in the flies and did not dramatically alter gene expression, it can significantly enhance the manifestation of *cbs* and *cse* deletions at the transcriptional and morphological levels. 

The generated double knockouts of the genes involved in H_2_S synthesis and the TSP resulted in severe disruption of homeostasis, which affected practically all the vital processes in the *Drosophila* organism, including lifespan, excretion, and reproduction. Thus, our study represents a significant step towards understanding the role of genes involved in H_2_S synthesis and metabolism in various life processes, including lifespan as well as reproduction. The lessons learned from the developed *Drosophila* model could be used to develop H_2_S-based therapeutics for various human diseases.

## Data Availability

The original contributions presented in this study are included in the article and Supplementary Materials.

## Supplementary Materials

The following supporting information can be downloaded at: https://www.mdpi.com/article/10.3390/antiox14060693/s1, Figure S1: Real-time PCR confirming the knockouts of genes (*cbs*, *cse*, and *dtst1)* in control *58492* and KO strains with deleted genes *cbs*, *cse*, *dtst1*, double KO (*cbs-/-*; *cse-/-*), and triple KO (*cbs-/-*; *cse-/-*; *dtst1-/-*); Figure S2: Standard dilutions of NaHS; Figure S3: Heatmap depicting LogFC values of genes comprised in three groups—UDP glucostransferases, glutathione S-transferases, and cytochromes; Figure S4: Heatmap depicting averaged values of LogFC among KEGG metabolic pathways; Figure S5: Heatmap of differentially expressed genes involved in the KEGG pathway “DNA repair”; Figure S6: The pattern of fecundity rate fluctuations during the lifespan of the compared strains. Figure S7: Common Gene Ontology terms for *cbs*, *cse*, double KO, and triple KO strains; Figure S8: MTs from strains *58492*; double KO, triple KO, and *dtst1-/-*; Figure S9: Comparative analysis of active center of rhodanese domains; Figure S10: Box diagram of expression levels of genes with rhodanese domain: *CG12279*, *CG4456*, *CG6000*; Figure S11: Heatmap of differentially expressed genes involved in a group of genes belonging to the ecdysteroid kinase family.; Table S1: Primers for QRT-PCR; Table S2: The measurement of lifespan parameters; Table S3: Comparison of reproductive period duration, offspring number and mortality of transgenic strains; Table S4: Deletion of *dtst1* leads to enrichment mainly in the metabolism of organic cyclic compounds; Table S5: GSEA analysis applied on genes differentially expressed both in double and triple KO transformants in ovaries; Table S6: GSEA analysis applied on reproduction-related genes, differentially expressed both in double and triple KO transformants in ovarian tissues.
