# Supplementary material for: The Biological Consequences of the Knockout of Genes Involved in the Synthesis and Metabolism of H2S in Drosophila melanogaster
Source: Antioxidants (Basel). 2025 Jun 6;14(6):693. doi: 10.3390/antiox14060693 (PMC12189691; doi:10.3390/antiox14060693)
Supplement: Supplementary file 1 [file antioxidants-14-00693-s001.zip › Supplements (1).pdf]

The biological consequences of the knockout of genes involved in the synthesis and metabolism of H_2S in *Drosophila melanogaster*

Victoria Y. Shilova [1], David G. Garbuz [1], Lyubov N. Chuvakova [1], Alexander P. Rezvykh [1], Sergei Y. Funikov [1], Artem I. Davletshin [1], Svetlana Y. Sorokina [2], Ekaterina A. Nikitina [3,4], Olga Gorenskaya [1,5], Michael B. Evgen'ev [1,6] and Olga G. Zatsepina [1,*]

Supplementary materials

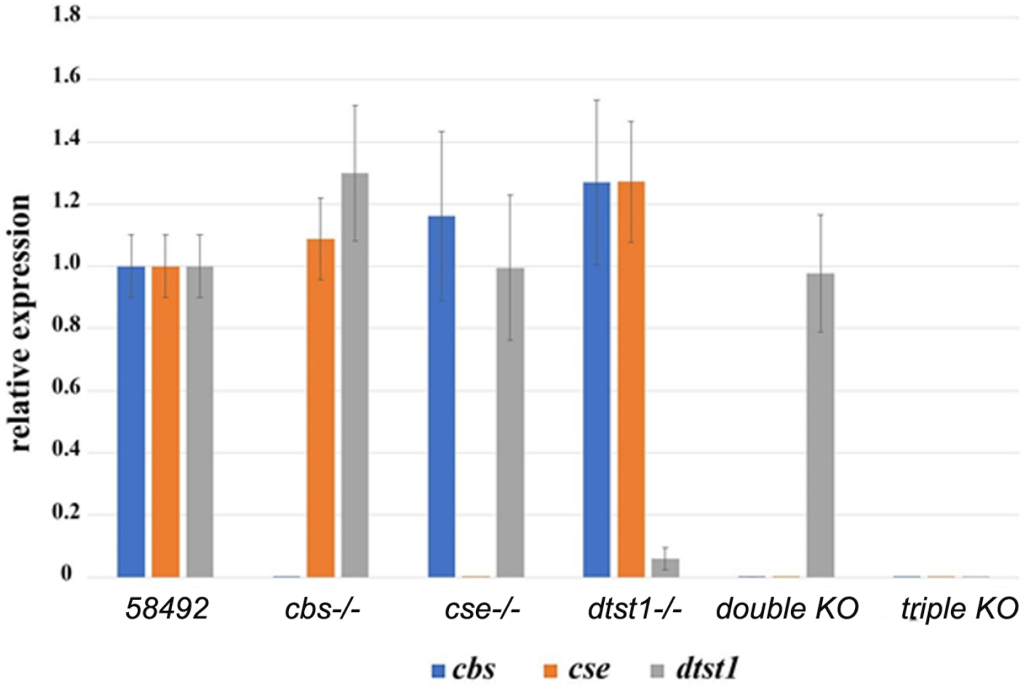

Figure S1.

Real-time PCR confirming the knockouts of genes (*cbs, cse,* and *dtst1)* in control *58492* and KO strains with deleted genes *cbs*, *cse, dtst1*, double KO (*cbs*-/-; *cse*-/-), and triple KO (*cbs*-/-; *cse*-/-; *dtst1*-/-)

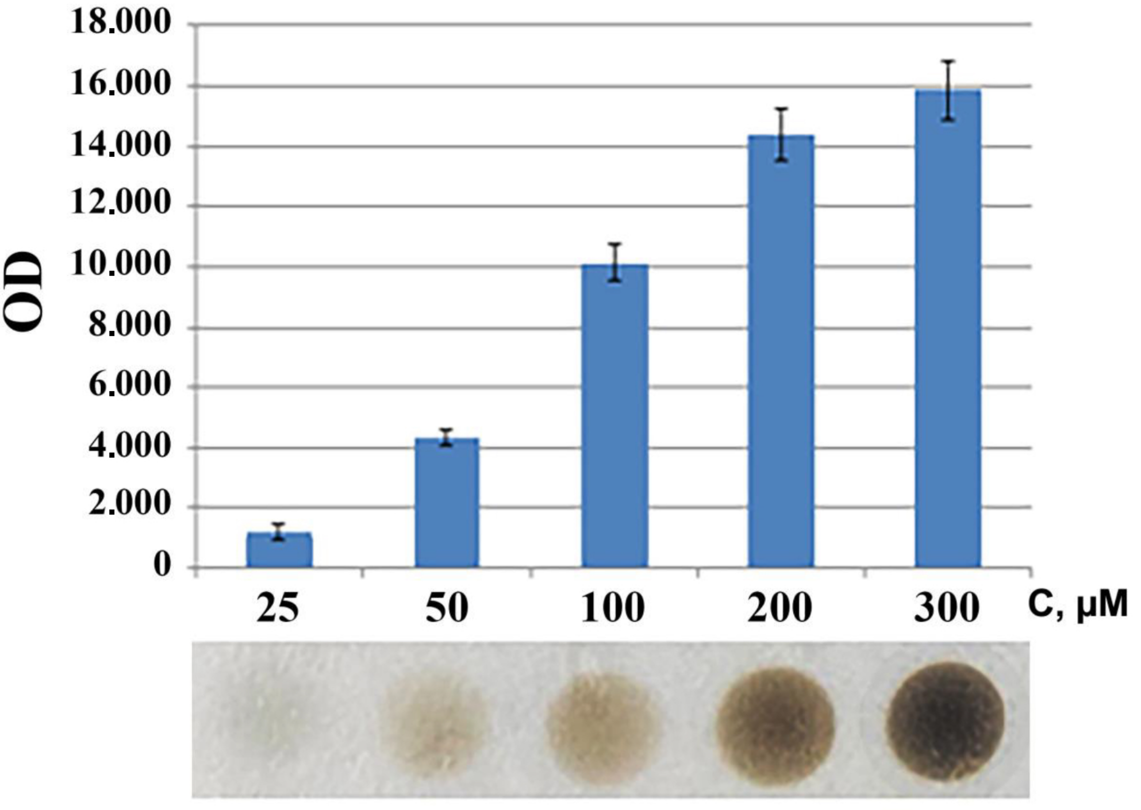

Figure S2. Standard dilutions of NaHS

Serial dilutions of NaHS for visualization of H_2S production. Dark cycles indicate the formation of lead sulfide on the paper. Calibration was carried out using NaHS (25 – 300 μM) dissolved in water. Circles on lead acetate paper after 20 h incubation at 30°C. Optical density was quantified using ImageJ software.

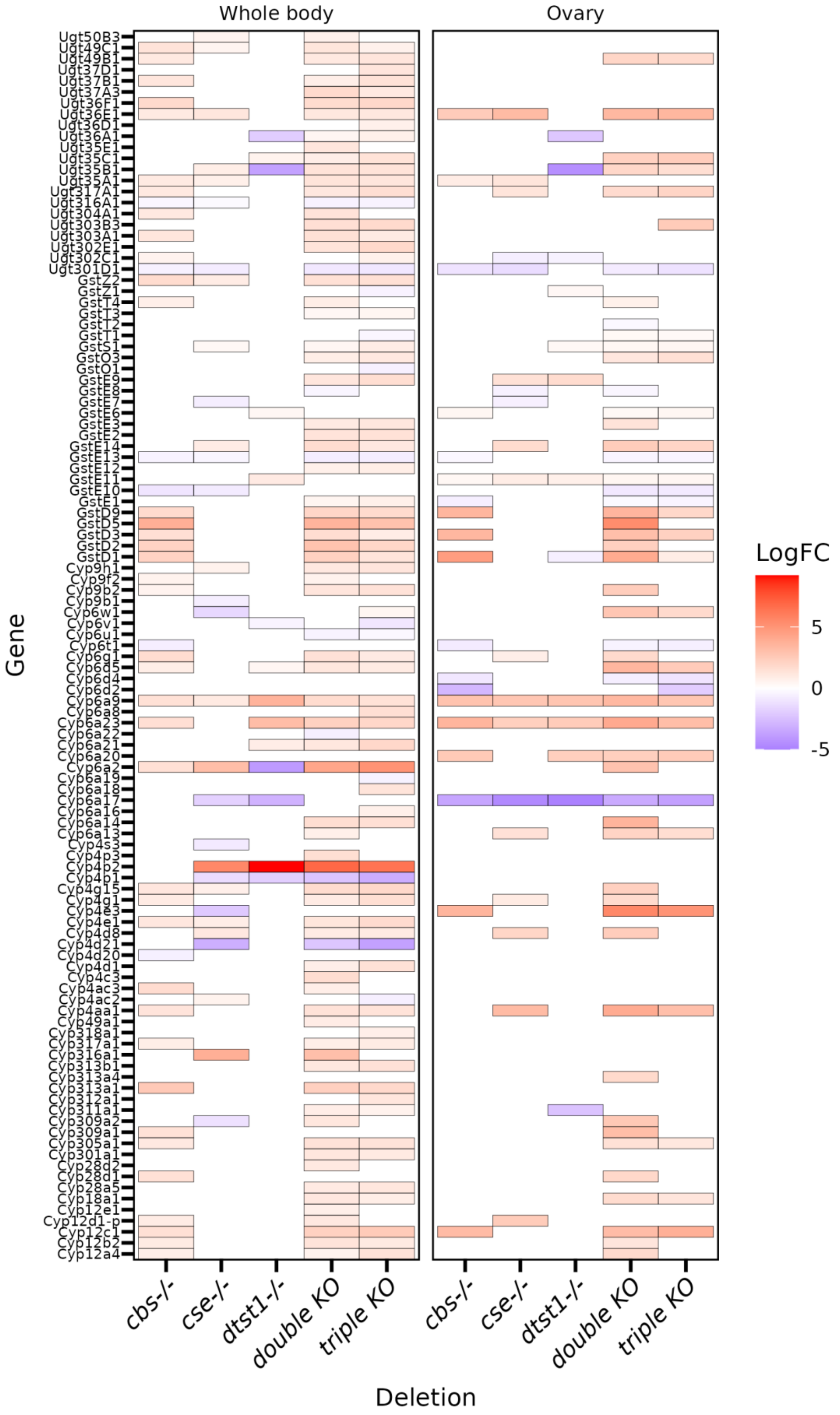

Figure S3. Heatmap depicting LogFC values of genes comprised in three groups - UDP glucostransferases, glutathione S-transferases, and cytochromes. Pairwise comparisons relative to the control strain are shown. Red positive log fold-change (log2FC). Blue, negative log2FC.

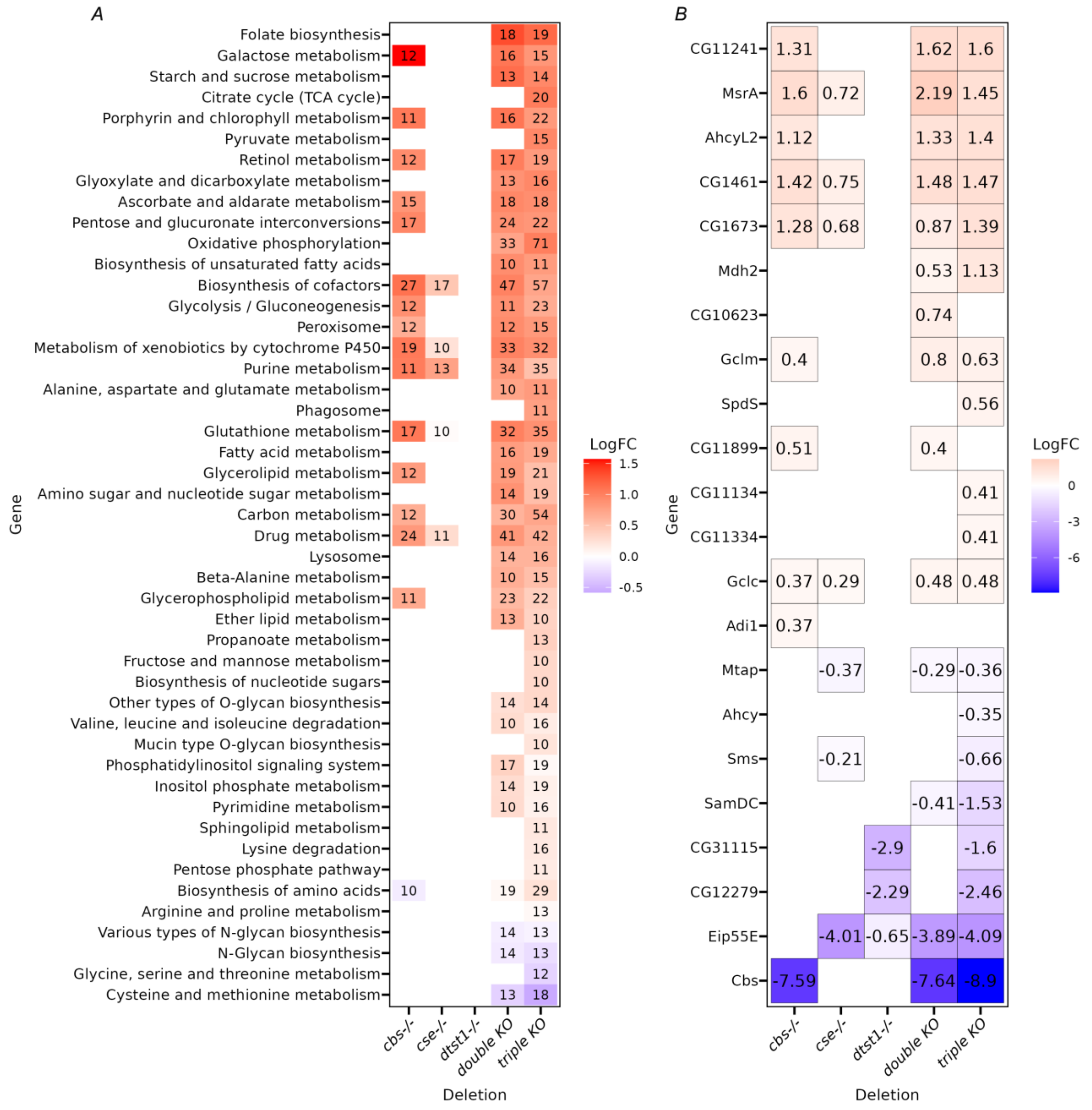

Figure S4. A - heatmap depicting averaged values of LogFC among KEGG metabolic pathways in all knockout fly strains. The number in each tile represents the number of differentially-expressed genes in each pathway. B - heatmap of DEGs on KEGG “Sulfur metabolism” pathway.

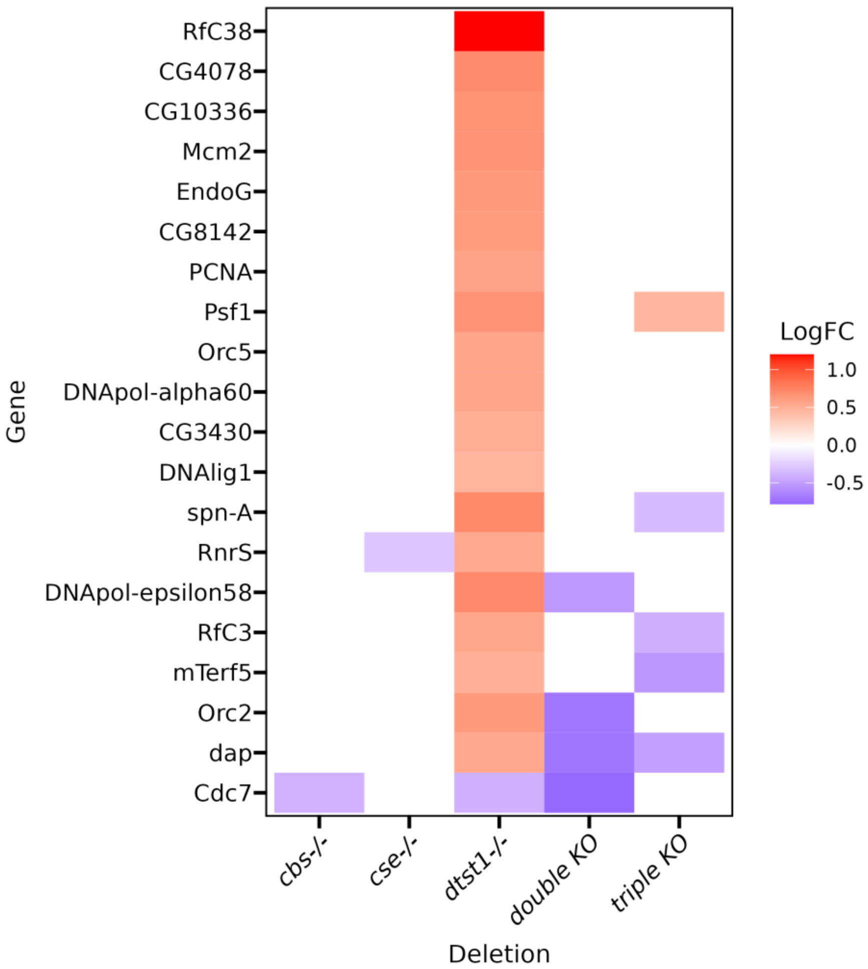

Figure S5. Heatmap of differentially expressed genes involved in the KEGG pathway "DNA repair". Pairwise comparisons relative to the control strain are shown. Red positive log fold-change (log2FC). Blue, negative log2FC.

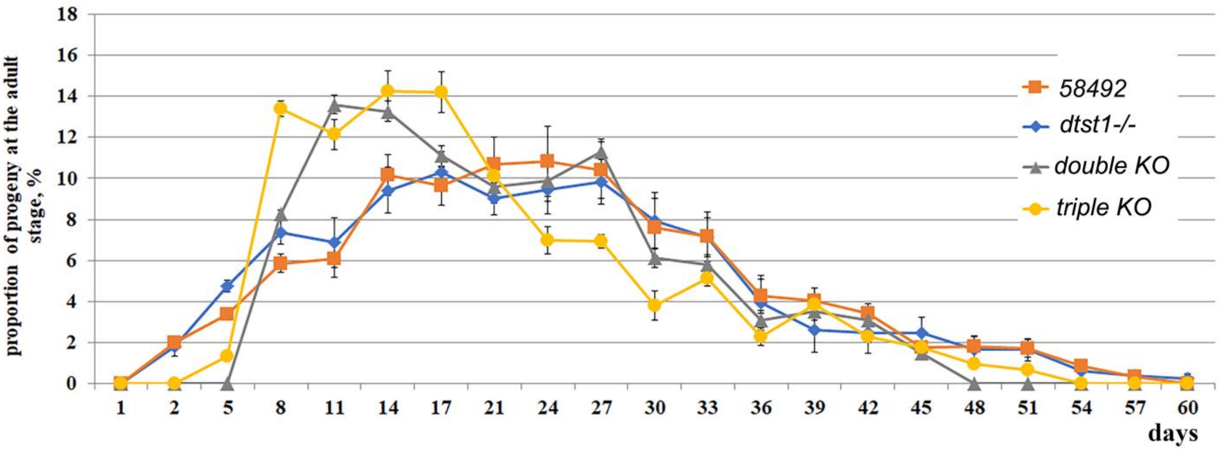

Figure S6. The pattern of fecundity rate fluctuations during the lifespan of the compared strains. $*p < 0.05$ significance was determined by two-way ANOVA followed by post-hoc Tukey's HSD tests.

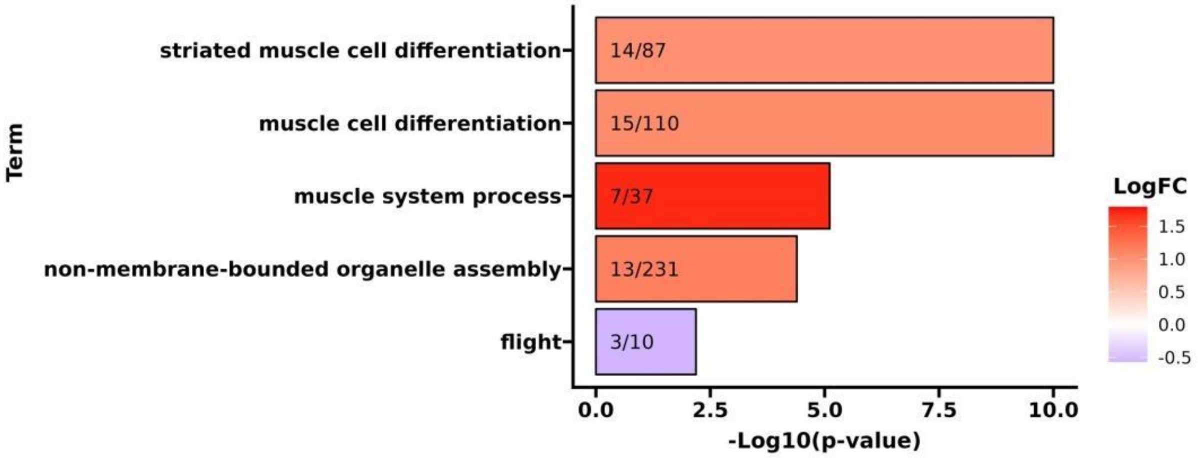

Figure S7. Common Gene Ontology terms for *cbs*, *cse*, double KO, and triple KO strains. The colored map shows averaged values of LogFC among KEGG for striated muscle cell differentiation, muscle system process, and membrane-bounded organelle assembly.

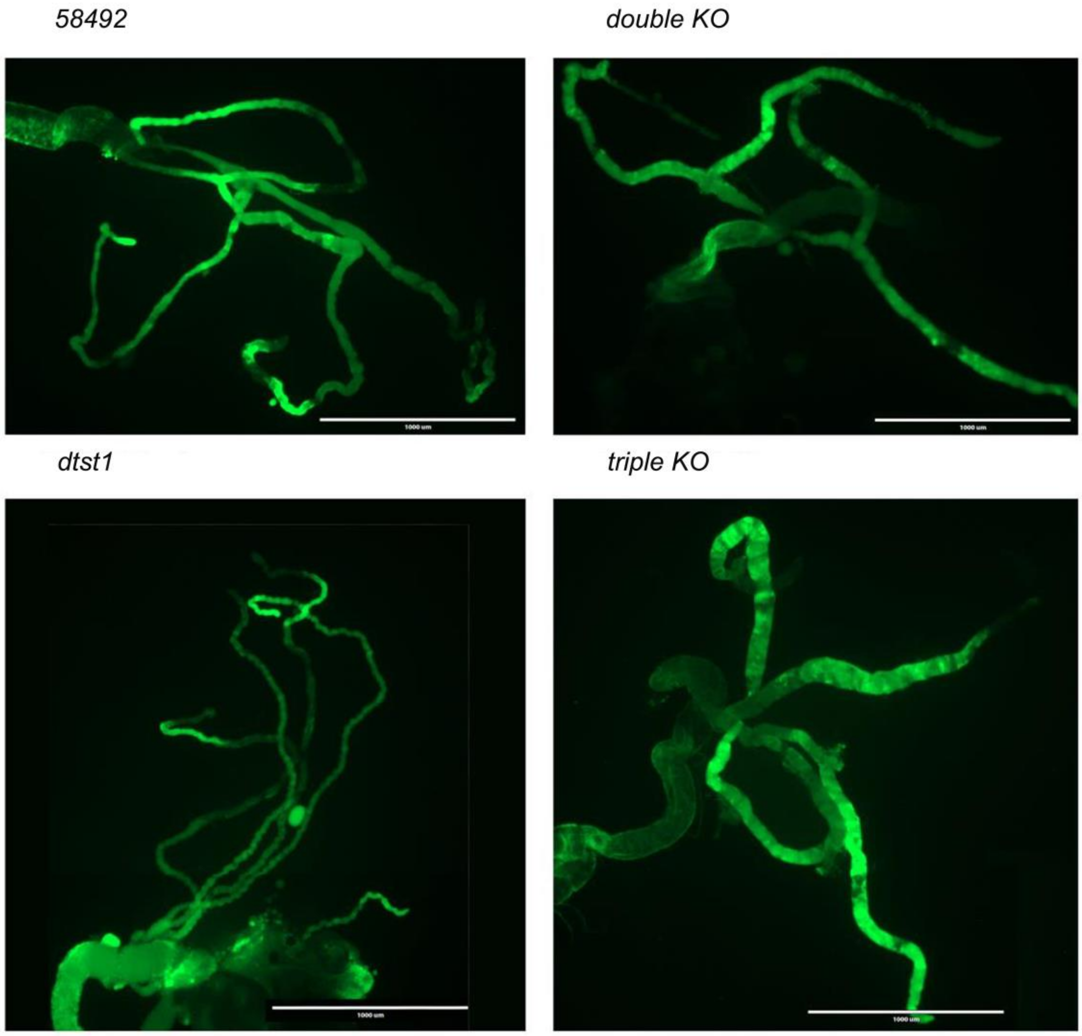

Figure S8. MTs from strains *58492*; double KO, triple KO, and *dtst1-/-*, were incubated with 2.5 μM SF7-AM in PBS for 30 min for live-cell imaging. Photographed on an Evos FL microscope at 4x equal magnification. Scale bar 1000 μm

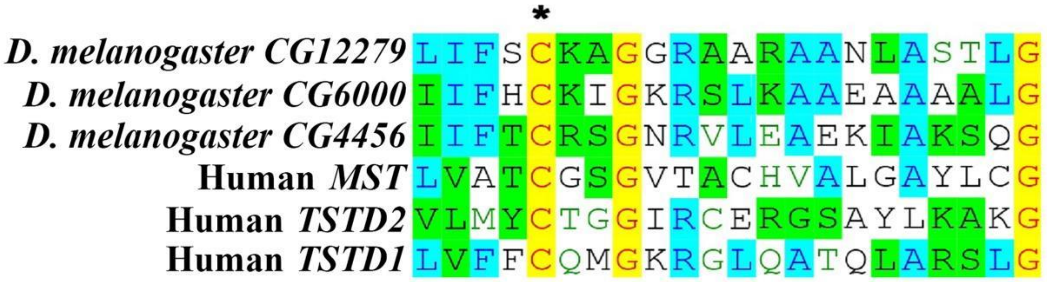

Figure S9. Comparative analysis of active center of rhodanese domains of *Drosophila melanogaster CG12279*, *CG4456*, *CG6000* genes and human *MST*, *TSTD2*, *TSTD1* genes belonging to the same family.

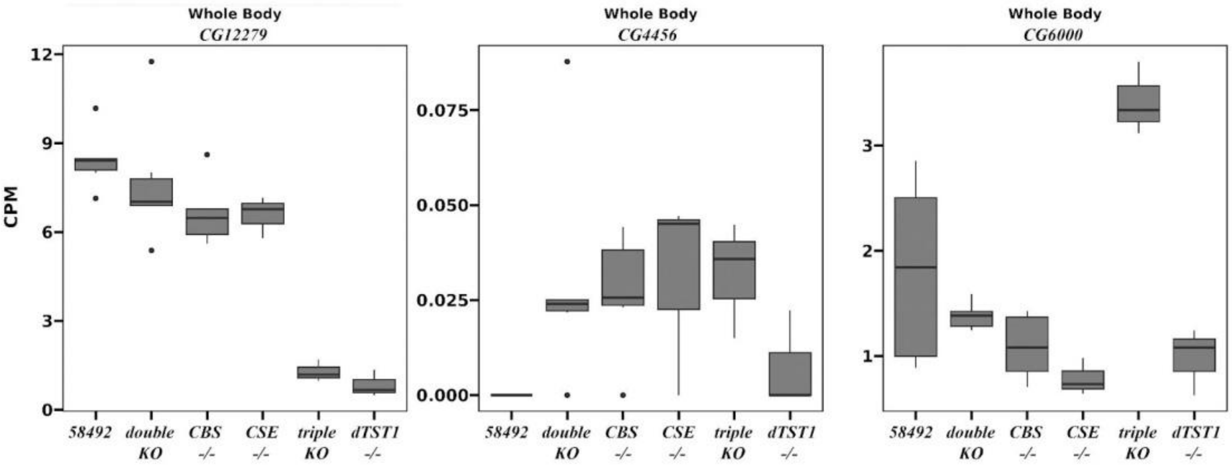

Figure S10. Box diagram of expression levels of genes with rhodanese domain: *CG12279*, *CG4456*, *CG6000* in *D. melanogaster* females from the studied strains.

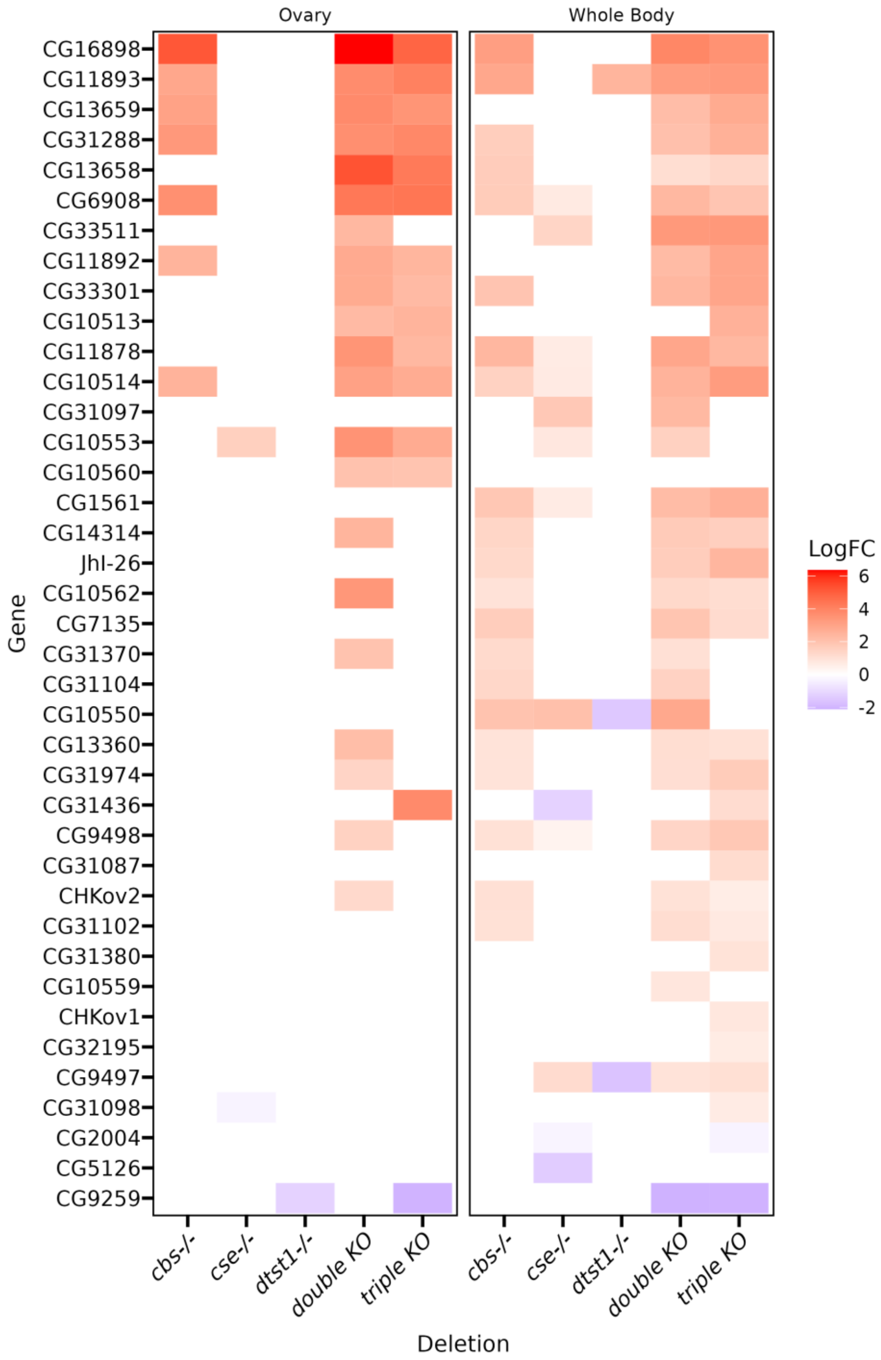

Figure S11. Heatmap of differentially expressed genes involved in a group of genes belonging to the ecdysteroid kinase family. Pairwise comparisons relative to the control strain are shown. Red positive log fold-change (log2FC). Blue, negative log2FC.

Primers for QPCR	Sequence
***rp49* FWD**	**5’-ATGCTAAGCTGTCGCACAAAT-3’**
***rp49* REV**	**5’-GTTCGATCCGTAACCGATGT-3’**
***cbs* FWD**	**5’-GCCAGCAGATTACGCCCAACA-3’**
***cbs* REV**	**5’-TAAGGAACTCGCACTTGGCAT-3’**
***cse* FWD**	**5’-CAACACCTTCCTGACCTCCTA-3’**
***cse* REV**	**5’-CGAATGGAGACGGCACAATG-3’**
***dtst1*FWD**	**5’-GGAGGAGGACTTCGCCCAG-3’**
***dtst1* REV**	**5’-ATCGGACGCAGTTGGGGTG-3’**

Table S1. Primers for QRT-PCR real time PCR confirming the knockouts of genes (*cbs*, *cse* and *dtst1*)

Name	N	Mean lifespan (LS).	50%	dM (%) 58492 LR	dM (%) double KO LR	90%	d90 (%) 58492 MW	d90 (%) double KO MW	100%	Mean LS of last 10% survivors
58492	296	56.96 ±0.88	61	-	-	69	-	-	75	71.09±1.02
dtst1-/-	419	62.96 ±0.75	68	11.5 P<0.001	28.3 P<0.001	76	11.0 P<0.001	22.6 P<0.001	90	81.09±1.11
Double KO	454	50.37 ±0.57	53	-8.9 P<0.001	-	62	-10.1 P<0.001	-	71	66.18±0.60
Triple KO	464	50.85 ±0.62	54	-11.5 P<0.001	1.9 P>0.05 (0.2305)	64	-7.2 P<0.001	3.2 P>0.05 (0.216)	73	67.5±0.61

Table S2: The measurement of lifespan parameters.

N – No of individuals; 50% (median), 90% and 100% – Age in days at % of mortality; dM (%) and d90 (%) – differences between median lifespan and age of 90% mortality of control (58492 or double KO) and experimental flies; LR – log Rank test with Bonferroni correction; MW – Mann-Whitney U test.

Drosophila **strain**	**days**	**Difference (%)**
58492	57	-
dtst1-/-	60	5
double KO	48	< 16
triple KO	51	< 11

A

Drosophila **strain**	**offspring number**	**Difference (%)**
58492	549	-
dtst1-/-	611	> 13
double KO	226	< 59
triple KO	174	< 68

B

Drosophila **strain**	**mortality from egg to pupae stage (%)**	**mortality from pupae to imago stage (%)**
58492	21	6
dtst1-/-	9	7
double KO	19	6
triple KO	34	13

C

Table S3. Comparison of reproductive period duration, offspring number and mortality of transgenic strains

A. Comparison of reproductive period duration of transgenic strains with control: double and triple KO strains demonstrate shorter reproduction period (by 22 and 16%) compared to the control 58492 strain. B. Comparison of offspring number from one pair of flies of transgenic strains for the whole reproductive period with the control strain (percent difference). C. Mortality at different stages of development (percent difference). Mortality from egg to pupae stage was calculated as the percentage of pupae to the total number of eggs laid. Mortality from pupae to the imago stage was calculated as the percentage of imago to the total number of pupae.

Enrichment FDR	nGenes	Pathway Genes	Fold Enrichment	Pathways (click for details)
2.0E-02	101	2836	1.5	GO:1901360 organic cyclic compound metabolic proc.
2.9E-02	152	4922	1.3	GO:0065007 biological reg.

Table S4. Deletion of *dtst1* leads to enrichment mainly in the metabolism of organic cyclic compounds.

Table S5. GSEA analysis applied on genes differentially expressed both in double and triple KO transformants in ovaries.

Table S6. GSEA analysis applied on reproduction-related genes, differentially expressed both in double and triple KO transformants in ovarian tissues
